# Temporal Associations Between Thyroid Cancer Epidemiological Trends and Classification Revisions

**DOI:** 10.3390/cancers18213468

**Published:** 2026-10-27

**Authors:** Maria Trovato, Balamurugan Pandiyan, Silvia Martina Ferrari, Fausto Famà, Salvatore Benvenga

**Affiliations:** 1 Department of Clinical and Experimental Medicine, University of Messina, 98122 Messina, Italy; s.benvenga@live.it; 2 Department of Mathematics, University of Wisconsin-Whitewater, 800 W. Main Street, Whitewater, WI 53190-1790, USA; pandiyab@uww.edu; 3 Department of Clinical and Experimental Medicine, University of Pisa, 56126 Pisa, Italy; silvia.ferrari@unipi.it; 4 Department of Biomedical and Dental Sciences and Morphofunctional Imaging, University of Messina, 98122 Messina, Italy; ffama@unime.it

**Keywords:** thyroid carcinoma, papillary thyroid cancer, follicular thyroid cancer, epidemiological divergence, Chow test, piecewise segmented regression, Markov-switching model, overdiagnosis, histopathological classification

## Abstract

Background/Objectives: This study evaluated potential temporal associations between international histopathological classifications, staging updates, and the long-term divergence observed between papillary thyroid cancer (PTC) and follicular thyroid cancer (FTC) incidence trends. Applying an advanced statistical protocol comprising the recursive Supremum Chow Test, Piecewise Segmented Regression with Regime Switching (PSR-RS), and a non-linear Markov-Switching Intercept and Slope (MSIA) model, we analyzed population-level annual incidence rates (AIR) in the United States from 1975 to 2023. Methods: We implemented a two-pronged approach to identify and validate structural trend changes in incidence trends. Approach 1 tracked endogenous, data-driven incidence shifts, while Approach 2 tested the fixed calendar milestones corresponding to the official publication dates of major classifications. Crucially, this multistage analytical protocol was applied directly to the individual PTC and FTC series, as well as to the scale-independent log ratio, ln(PTC/FTC) and aggregate diagnostic gap timelines. Results: The results show that the incidence gap between the two cancers widened until 2014 and stabilized thereafter. AG quadrupled from 2.9 per 100,000 population in 1975 (PTC AIR = 3.6 minus FTC AIR = 0.7) to 11.6 in 2014 (PTC AIR = 12.8 minus FTC AIR = 1.2) with no further increase thereafter (AG = 11.6 in 2022 and 2023). Under Approach 1, the natural breakpoints partitioned the timelines into highly diverse behavioral segments. Specifically, the data identified five evolutionary regimes for PTC, three independent regimes for FTC, and four distinct regimes for AG. The breakpoint identified in 1987 coincided with the staging updates introduced that year and marked a clear change in the incidence trends of both histotypes. Between 1987 and 1991, PTC incidence increased, while FTC incidence declined toward its historical minimum. Despite these changes, the aggregate diagnostic gap continued along its existing trend, with no structural break detected. The 2004 WHO third edition corresponded to a systematic upward level shift (β_2_ = +4.1500) associated with the formal codification of microcarcinomas. Finally, both analytical approaches converged on 2014 as a significant historical turning point in the slowing of incidence growth. This phase is characterized by a significant negative slope change (Δβ = −0.35) consistent with conservative classification updates and subsequent stabilization at a structural plateau. Conclusions: The progressively widening AIR gap between PTC and FTC does not appear to reflect a purely biological phenomenon. Instead, these population-level changes are highly consistent with systematic reporting shifts and diagnostic thresholds introduced in successive international staging and histopathological classifications.

## Simple Summary

This study examined the potential explanations for the dramatic increase in the incidence of papillary thyroid cancer (PTC) over the past five decades, while the incidence of follicular thyroid cancer (FTC) remained relatively stable. Using advanced statistical methods, we analyzed annual incidence rates (AIRs) for PTC and FTC in the United States from 1975 to 2023. We evaluated changes in incidence over time and compared them with the publication dates of successive editions of thyroid cancer (TC) classifications. Our findings show that the substantial, widening gap in incidence between these two cancers is consistent with systematic shifts in classification and reporting rather than a purely biological explanation. New classification rules introduced in 1987 coincided with a shift in reported cases from FTC to PTC. Later, in 2004, revised criteria were followed by increased registration of very small tumors. Finally, updated guidelines introduced in 2014 adopted more conservative diagnostic thresholds, which coincided with a slowing of incidence growth and subsequent stabilization at a plateau.

## 1. Introduction

At the beginning of the third millennium, the Surveillance, Epidemiology, and End Results (SEER) program and the GLOBOCAN 2000 project of the International Agency for Research on Cancer (IARC) reported a gradual stabilization in incidence alongside reduced mortality rates for most malignancies in both the USA and Europe [1,2,3]. Conversely, contemporary epidemiological reports highlighted that incidence and mortality rates for thyroid cancer (TC) were increasing in these same geographic regions [4,5,6,7,8,9].

A quarter of a century later, this epidemiological landscape has partly changed in the USA. Based on annual percent change (APC) estimates, the SEER program documented a progressive decline in overall cancer incidence during the first decade of the new millennium, particularly among males (Table S1 in Supplementary Materials, left). Conversely, during the second decade, TI for cancers increased, particularly among females (Table S1 in Supplementary Materials) [2,10]. In the subsequent decade, however, the overall cancer incidence increased, particularly among females (Table S1 in Supplementary Materials) [2,10]. During the first 24 years of the current century, cancer mortality rates in the USA continued the steady decline observed in both sexes during the late twentieth century (Table S1 in Supplementary Materials) [11]. For TC specifically, incidence increased to a similar extent in both sexes during the first 14 years of the current century [11] (Table S1 in Supplementary Materials, right). In contrast, the TC incidence declined from 2013 to 2023, with a more pronounced reduction among females than males [11] (Table S1 in Supplementary Materials). Consequently, in a reversal of twentieth-century patterns, overall cancer incidence increased while TC incidence declined (Table S1 in Supplementary Materials). TC mortality rates increased only during the first decade, diverging from the patterns observed in the late twentieth century. Notably, across the three-time intervals summarized in Table S1 (Supplementary Materials), TC mortality showed distinct differences between the sexes, remaining stable among females while increasing among males.

The latest available international epidemiological data, compiled in the GLOBOCAN 2022 project, rank TC as the seventh-most common malignancies worldwide, with 821,214 new cases, and the 24th leading cause of cancer death globally, with 47,507 deaths [12,13]. These metrics reported in the IARC Cancer Factsheets, Global Cancer Observatory (GCO), and Cancer Today GLOBOCAN project represent the latest standardized population statistics [12,13]. The overall prognosis for TC remains favorable, showing progressive improved compared with the previous century [10,14]. In the USA, TC accounts for approximately 0.5% of all cancer deaths, with an overall 5-year survival rate exceeding 90% [10]. For subsequent annual frames, estimates indicated approximately 45,240 new TC cases in the USA, representing 2.1% of all newly diagnosed malignancies [15]. The estimated deaths due to TC were 2,320 (0.4% of all cancer deaths), while the 5-year relative survival rate reached 98.3% [15].

These epidemiological trajectories should be interpreted in light of the systematic modifications in clinical practice introduced to counteract overdiagnosis [10,16,17]. Recommendations advising a reduction in routine screening, alongside the adoption of more restrictive diagnostic criteria, represent prominent factors associated with the recent contraction in TC incidence [10,16,17]. However, a definitive assessment of the long-term impact of these clinical transformations on TC mortality remains limited because of the leveling of mortality rates observed over the past decade (Table S1 in the Supplementary Materials). Multiple investigations have emphasized that long-term trend analytics are essential for healthcare strategies, as they provide objective insights for evaluating the historical impact and population-level consequences of evolving medical practices [18,19,20].

The standardized recording of diagnoses within a population forms the foundation for tracking epidemiological trends. The annual incidence rate (AIR) of a disease is calculated as the ratio of new cases to the population at risk during a 12-month period, expressed per 100,000 or 1,000,000 individuals. Consequently, if the diagnostic criteria for a specific disease undergo modifications starting in a given calendar year, subsequent variations in the calculated AIR are mathematically expected. Clarifying the relationship between revisions to diagnostic criteria and AIR dynamics is crucial for informing public health policies and endocrine oncology strategies.

Currently, the definitive diagnosis of TC relies on histopathological examination, where morphological characteristics, including the embryological origin of thyroid cells, serve as the core criteria for tumor classification [21,22,23,24]. Thyroid malignancies arise from either hormone-producing follicular cells (thyrocytes) or calcitonin-producing parafollicular cells (C cells) [25,26,27,28]. Follicular cells give rise to follicular thyroid carcinoma (FTC), papillary thyroid carcinoma (PTC), and anaplastic thyroid carcinoma (ATC), whereas C cells give rise to medullary thyroid carcinoma (MTC) [25,26,27,28]. FTC and PTC are the most common histotypes of well-differentiated thyroid carcinoma (WDTC) [25,26,27,28]. Distinct morphological and architectural features distinguish FTC from PTC on microscopic examination, and the SEER program currently uses this distinction to collect population-level data in the USA [2].

Four international organizations have developed standardized TC classification and reporting frameworks: (i) the Armed Forces Institute of Pathology (AFIP), (ii) the World Health Organization (WHO), (iii) the International Union Against Cancer (UICC), and (iv) the American Joint Committee on Cancer (AJCC) [29,30,31,32]. A fundamental distinction must be made between the nature and operational objectives of these frameworks. Histopathological tumor classifications, such as those published by the AFIP and the WHO, focus primarily on defining microscopic histotypes and cellular criteria. The first historical histopathological classifications from these organizations were published by the AFIP in 1953 [25,33] and by the WHO in 1974 [34,35]. Conversely, staging classifications, such as those developed by the UICC and the AJCC, do not redefine microscopic criteria but focus on categorizing disease severity and anatomical extension via the Tumor (T), Node (N), and Metastasis (M) system to establish prognostic categories [36]. The UICC introduced the TNM framework in its first edition of its TNM Classification of Malignant Tumors in 1968, while the AJCC established its clinical staging systems in the first edition of the Manual for Staging of Cancer in 1977 [30,37,38,39]. In 1987, the UICC and AJCC unified their criteria to provide a synchronized international staging framework [30].

Building on these premises, the present study evaluates the historical classifications and staging revisions released from 1974 to 2023 to determine whether these international updates show specific temporal associations with variations in population-level epidemiological trends, particularly for FTC and PTC. Population data collection began in 1975, following the baseline histopathological criteria established by the 1974 international updates. Subsequent staging and histopathological revisions were systematically evaluated over the same historical timeframe. To achieve this objective, we first analyzed the AIR series for FTC and PTC from 1975 to 2023 to map their long-term trajectories and assess the progressive widening of the diagnostic gap. Second, utilizing an advanced time-series protocol based on three complementary statistical frameworks, we tested whether the endogenously identified trend breakpoints showed significant temporal associations with the publication dates of international staging and classification standards.

## 2. Materials and Methods

All TC classifications described in the Introduction were reviewed, taking note of the number of editions, publication years, and average publication years.

For the historical timeframe spanning 1975 to 2023, population-level incidence data were retrieved from the SEER Program database (National Cancer Institute, Bethesda, MD, USA), specifically utilizing the SEER*Stat Database: Incidence - SEER Research Data, 21 Registries, November 2025 Submission (2000–2023), released in April 2026. Statistical analyses and data extraction were performed using SEER*Stat software version 9.0.43 (Surveillance Research Program, National Cancer Institute, Bethesda, MD, USA; available at cancer.gov) [1,2]. The database was filtered using the SEER Site Recode ICD-O-3/WHO 2008 variable, restricted specifically to the thyroid site. The extracted dataset encompasses both sexes and includes the total aggregate population without distinctions by race to guarantee an unselected, population-level baseline representation. The diagnostic confirmation variable was restricted to microscopically confirmed cases. The PTC cohort encompasses International Classification of Diseases for Oncology (ICD-O-3) morphology codes 8050, 8052, 8260, 8340 (follicular variant of PTC), 8341 (papillary microcarcinoma), 8342, 8343, and 8344 [1,2]. Conversely, the aggregate FTC Histology Type encompasses ICD-O-3 morphology codes 8290 (oxyphilic adenocarcinoma/Hürthle cell carcinoma), 8330, 8331, 8332, and 8335 [1,2]. Due to historical registry reporting specifications, these sub-morphologies were evaluated within their aggregate histological definitions over the 49-year continuous timeline. TC incidences were provided by AIR for both FTC and PTC as recorded in the SEER [1,2]. On this website, the AIR is given per 100,000 USA population and age-adjusted to the 2000 USA standard population [1,2].

The time series of AIR for both PTC and FTC were investigated to characterize their temporal dynamics, structural shifts, and the evolving diagnostic gap over time. Descriptive and inferential statistics were performed to characterize the baseline properties of the individual PTC and FTC series. Descriptive analysis included minimum (min), maximum (max), sample mean (M), median (Mdn), standard deviation (SD ±), and variance (VAR). To formally assess the distributional configuration of the incidence rates and verify the compliance of the follicular baseline with a Gaussian structure, a formal Shapiro–Wilk normality test (W) was performed across the continuous timeline.

Inferential statistical association was evaluated via covariance (Cov), Spearman coefficient (Rho), and Pearson correlation coefficient (r). To prevent spurious associations driven by shared chronological trends and temporal autocorrelation, association metrics were further validated by calculating the Pearson correlation coefficient on first-differenced, detrended series (rΔ).

To detect endogen breakpoints inside historical AIR time series for FTC and PTC, a protocol was developed. The protocol relies on the use of three established models used to complement each other: a standard linear regression model (Chow Test) and two segmented regression models, namely, Piecewise Segmented Regression with Regime Switching (PSR-RS) and Markov-Switching intercept and slope (MSIA) [40,41,42,43,44,45,46,47,48].

For the data-driven timeline evaluation (Approach 1), candidate breakpoints were selected via an objective, automated statistical algorithm to ensure complete mathematical reproducibility and eliminate subjective visual inspection. This screening was performed via a recursive Supremum Chow Test (Quandt–Andrews framework) implemented in R software version 4.4.1 (R Foundation for Statistical Computing, Vienna, Austria; available at https://www.R-project.org/) utilizing the standard ‘strucchange’ package version 1.5-3 (Achim Zeileis et al., Vienna University of Economics and Business, Vienna, Austria; available at https://CRAN.R-project.org/package=strucchange) [49]. The algorithm systematically scans the continuous series and computes the F-statistic by comparing the pooled Residual Sum of Squares (RSS_C) against the partitioned sub-period residuals (RSS_1_ and RSS_2_) across every consecutive year: F = [(RSS_C − (RSS_1 + RSS_2))/k]/[(RSS_1 + RSS_2)/(n_1 + n_2 − 2k)]. The year maximizing this test statistic identifies the endogenous rupture point [41]. To strictly control for multiplicity inflation and minimize Type I error rates across the simultaneous testing matrices, the family wise error rate was adjusted by applying the Benjamini–Hochberg False Discovery Rate (FDR) correction across all 39 concurrent equations under standard significance boundaries (α = 0.05).

Subsequently, the structural parameters were evaluated using the PSR-RS and MSIA frameworks. The PSR-RS model extends linear trend analysis by incorporating a continuous time trend and a binary indicator variable to capture both the immediate level adjustment and trajectory change at a predefined milestone (T*): Y_t_ = β_0_ + β_1_T_t_ + β_2_D_t_ + Δβ(T_t_ − T*)D_t_ + ε_t_ where D_t_ is a binary dummy variable setting to 1 for t ≥ T* [42,43]. To eliminate the bias of inflated test statistics caused by residual serial correlation and non-stationarity, all PSR-RS model parameters, including the level change coefficient (β_2_) and slope modification coefficient (Δβ), are estimated utilizing Newey–West Heteroscedasticity and Autocorrelation Consistent (HAC) standard errors, with the lag truncation parameter objectively selected based on automatic bandwidth selection.

Unlike the segmented linear framework, the nonlinear MSIA model does not rely on a fixed calendar breakpoint. Instead, the parameters vary according to an unobserved latent state (S_t_) governed by a first-order Markov chain, functioning as an automated endogenous scanning tool. For a two-state model (S_t_ ∈ {1, 2}), the formulation is expressed as Y_t_ = β_0_(S_t_) + β_1_(S_t_)T_t_ + ∑_i=1_ᵖ φ_i_ Y_t−i_ + ε_t_(S_t_) [48].

The MSIA model utilizes an automated Expectation-Maximization (EM) algorithm to compute smoothed state transition probabilities linked to the data’s internal dynamics. A clear distinction is made regarding how these models evaluate regime levels, as the parameters capture different statistical properties. In the PSR-RS framework, β_2_ represents a deterministic vertical adjustment isolated at a single, fixed point in time (T*) [42,43]. Conversely, the MSIA model captures level changes through state-dependent intercepts β_0_(S_t_) combined with an autoregressive component (∑_i=1_ᵖ φ_i_ Y_t−i_) [47,48]. Because MSIA processes variations via probabilistic transition matrices, its intercept values reflect the smoothed average level of a latent state across the continuous timeline rather than a localized adjustment. Therefore, β_2_ from PSR-RS and the intercept dynamics of MSIA operate under different mathematical specifications and will inherently yield distinct numerical values. To maintain structural alignment during the comparative phase, the endogenous parameters of the exploratory MSIA model are handled as the implicit intercept shift (computed as β_0_,_2_ − β_0_,_1_) and the implicit slope differential (computed as β_1_,_2_ − β_1_,_1_), which serve as the direct counterparts to the deterministic β_2_ and Δβ of the PSR-RS specifications, respectively [47,48]. Both measurements are crucial for disentangling the dynamic nature of regime switching. Indeed, the PSR-RS framework isolates the immediate, short-term impact, that is, the “announcement effect” or structural break captured by β_2_; the MSIA framework determines the long-term persistence and endogenous sustainability of the new regime. Together, they allow for a comprehensive assessment of whether a historical event causes an abrupt structural rupture or triggers a gradual evolutionary transition within historical time series.

By combining these three distinct approaches, our protocol establishes a rigorous multi-stage validation framework. The initial a priori hypothesis tested using the Chow Test is corroborated independently by the deterministic structural parameters of the PSR-RS and further cross-validated by the probabilistic state-transitions of the MSIA model. This integrated strategy ensures that the identified structural breaks represent genuine regimes rather than statistical artifacts of the selected timeline.

## 3. Results

### 3.1. Creation of Timeline: From the Inception of TC Classifications to Their Progress

To establish a reproducible chronological baseline, the official platforms of the AFIP/ARP, WHO, UICC, and AJC/AJCC were systematically examined to isolate the primary milestones of their respective reporting systems [29,30,31,32,35]. Detailed documentation was compiled regarding the total number of editions and exact publication years for the AFIP Atlas of Tumor Pathology [33,50,51,52,53], the WHO Histological Classification of Thyroid Tumours [34,35,54,55,56], the UICC TNM Classification of Malignant Tumours [36,57,58,59,60,61,62,63,64], and the clinical staging standards within the AJC/AJCC Manual for Staging of Cancer [37,65,66,67,68,69,70,71] (Table 1).

#### 3.1.1. AFIP/ARP Classifications

The historical record of the AFIP spans from its initial reporting baseline in 1953 to its formal transition in 2011 [32]. After the AFIP disestablishment, a new federal government pathological center, namely “the Joint Pathology Center”, was activated [32]. The AFIP established the earliest historical histological classification for thyroid malignancies, introduced in fascicle 14 of the first AFIP Series authored by Warren and Meissner (Table 1) [25,33]. In this initial text, medullary thyroid carcinoma (MTC) was not classified as a distinct histotype; instead, these lesions were categorized within the broader group of neuroendocrine malignancies before the formal nomenclature of MTC was proposed by Hazard et al. in 1959 [72].

From 1953, the AFIP staff took an active part in the standardization of an international medical nomenclature for neoplastic diseases through histological classification of cancers. The American Registry of Pathology (ARP) verified the AFIP diagnostic criteria in a very large number of tumor specimens: more than 3 million archival paraffin-embedded cases and over 10,000 frozen tumor specimens dedicated exclusively to molecular and genetic research [32,73,74].

**Figure 1:**
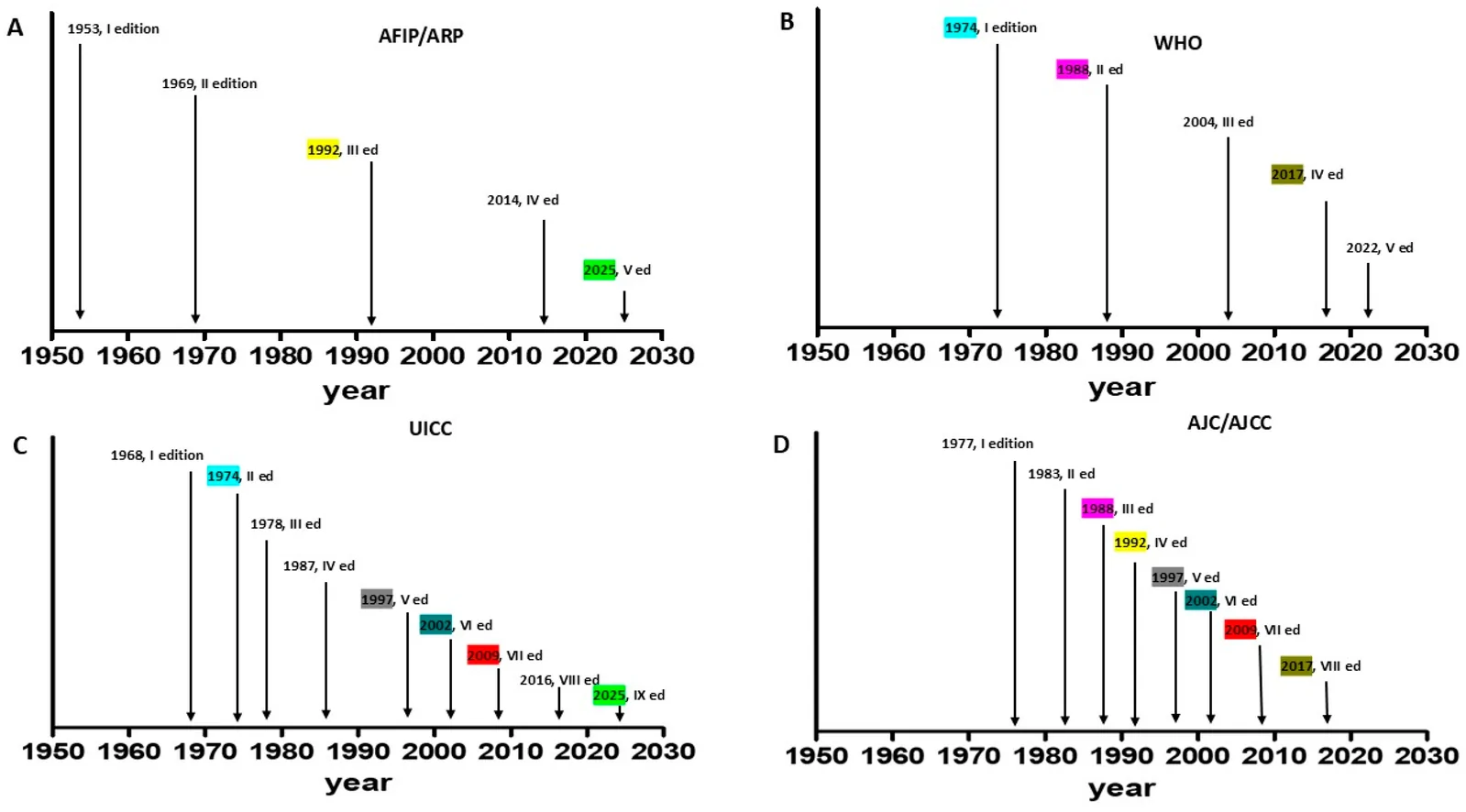
Timeline for each thyroid cancer classification. Footnotes: Panels show the chronological publication years for classification guidelines by (**A**) Armed Forces Institute of Pathology/American Registry of Pathology (AFIP/ARP), (**B**) World Health Organization (WHO), (**C**) Union for International Cancer Control (UICC), and (**D**) American Joint Committee on Cancer (AJCC/AJCC). Arrowheads indicate the specific publication year for each respective edition. Roman numerals designate the specific edition number released in each indicated year. The colors highlight the specific years in which multiple classification systems were published simultaneously.

Based on this patho-clinical foundation, the AFIP and ARP published a continuous sequence of thyroid tumor pathology fascicles (Table 1 and Figure 1A). Following the reorganization of the primary facility, updated tumor atlases have been published through independent ARP initiatives [32]. In summary, the combined AFIP and ARP organizations released five distinct series over a 73-year timeframe, maintaining a mean distribution interval of 18.0 years between consecutive publications (Table 1 and Figure 1A).

#### 3.1.2. WHO Classifications

In 1958, the WHO promoted an international program to launch a new nomenclature for the classification of tumors [31]. To standardize histological diagnoses of cancers, the WHO introduced a modern method for arranging denominations of cancers [31]. Ultimately, this was an international language codified on the basis of histological indicators used for diagnosing tumors [31]. However, the WHO histological biomarkers are not merely diagnostic tools. By incorporating tumor topography and morphology, the WHO classifications also aim to indicate cancer behavior [31]. Currently, the WHO remains a coding system to distinguish benign from malignant neoplasms. The first WHO histological classification of TC was published in 1974 (Table 1 and Figure 1B) [34]. From 1974 to date, the WHO has supplied five successive editions of the Classification of Endocrine and Neuroendocrine Tumors [31]. Therefore, in about 48 years, five successive WHO histological classifications have appeared at an average interval of 12.0 years (Table 1 and Figure 1B) [31].

A relevant development within the WHO TC reporting system involved incorporating specific molecular alterations into histological features [56]. The objective was to refine histological diagnoses by using a system in which distinct molecular indicators were linked to benignity or malignancy. The fifth edition of the WHO Classification of Endocrine and Neuroendocrine Tumors, published in 2022, features two changes in the diagnosis and management of thyroid tumors [23,75]. First, the classification introduced the category of “risk neoplasms”; second, molecular alterations became an integral component of the diagnostic process [23,75]. Therefore, on the basis of molecular profiles, TC are distinct in two distinct molecular “variants” named BRAF-like and RAS-like, respectively [23,75].

#### 3.1.3. UICC Classifications

The UICC was founded in 1933 to confront cancerous diseases through international medical cooperation [29,76]. To study the basis of an anatomo-clinical classification of tumors was one of the primary UICC scientific goals [29,76]. In 1936, Dr. Gustave Roussy became the first head of the commission responsible for the compilation of this type of classification [29,76]. However, only after the Second World War, Prof. Pierre Denoix harmonized the data collected from international reports on tumors [29,76,77]. In 1950, he launched a large-scale project for the nomenclature of cancers through three conventional indicators which are widely known by their abbreviations: T, N, and M. He evaluated the size and extension of primary tumors (T) simultaneously with the absence or detection of lymph node involvement (N) as well as with the absence or presence of metastatic spread (M) [29].

This project was completed in 1968 with the publication of the first categorization of cancers, known as the tumor-node-metastasis (TNM) classification, in the form of a pocket book titled Livre de Poche. (Table 1 and Figure 1C) [29,76]. Initially, the TNM system was adopted for 23 human organs, including TC [78,79]. From 1968 to date, UICC has published nine editions of the TNM Classification of Malignant Tumours at a mean interval of 7.1 years between subsequent editions (Table 1 and Figure 1C).

#### 3.1.4. AJC/AJCC Classifications

The AJCC was founded in 1980 following the antecedent AJC, which had been established in 1959 [30,38,39]. Both parent organizations shared the same agenda of developing a system of clinical staging for cancers to be adopted in medical practice across the USA [30,38,39]. This system had to be consistent with other accepted classifications. Staging schemes were set up by presuming that in a specific organ, cancers showing similar histological features have interchangeable patterns of growth and quite similar extension [30,38,39].

From 1962 until 1974, the AJC printed its cancer staging schemes in individual fascicles [see Introduction to the III edition of the “Manual for Staging of Cancer” [30,39,66]].

The AJC stages were related to the sites of cancerous occurrence. In 1977, the AJC published its first and only edition of the Manual for Staging of Cancer (Table 1) [79,80]. In 1983, the AJCC edited the second edition of the Manual for Staging of Cancer (Table 1 and Figure 1D) [30]. In 1987, the UICC and AJCC definitions were unified into the UICC TNM Classification of Malignant Tumours book (Table 1 and Figure 1D) [30].

The outcomes of this collaboration between the UICC and AJCC were documented in the third edition of the AJCC Cancer Staging Manual and the fourth edition of the UICC TNM Classification of Malignant Tumors [81]. From 1977 to the present, eight editions of the Manual for Staging of Cancer were released at a mean interval of 7.1 years between subsequent editions (Table 1). A major task in aligning the UICC and AJCC classification systems was to reliably predict patient outcomes through a unified clinical language. The TNM categories alone can be insufficient to fully gauge cancer severity, as patient outcomes depend not only on tumor anatomy but are also heavily influenced by clinical and host variables, such as age, sex, ethnicity, comorbidities, and the efficacy of chemotherapy. Recognizing that the TNM system cannot encompass all these clinical and treatment-related factors, the UICC and AJCC have focused on refining anatomical staging while encouraging the integration of these independent clinical variables through separate prognostic models [39].

However, clear differences emerged between the UICC and AJCC categorizations 30 years later. Indeed, these discrepancies formally became known with the publication of the UICC and AJCC 8th editions in 2016 and 2017, respectively [63,71]. These differences stemmed from two fundamentally contrasting paradigms that prioritize anatomical extension versus biological behavior. The UICC maintains that cancer staging must remain strictly anatomical, relying solely on the physical footprint of the disease: the size and depth of the primary tumor (T), regional nodal involvement (N), and distant metastasis (M). This conservative approach guarantees universal utility, ensuring that healthcare providers in low- and middle-income countries can accurately stage patients without the burden of expansive molecular or genetic testing. Conversely, the AJCC transitioned from a purely descriptive anatomical system to a highly dynamic prognostic model. The AJCC contends that traditional, anatomy-only TNM parameters are no longer sufficient to predict clinical outcomes or guide modern tailored therapies. Consequently, they directly integrate non-anatomical biological variables—such as tumor grade, receptor status, and genomic profiles—into the official staging calculus [82].

**Figure 2:**
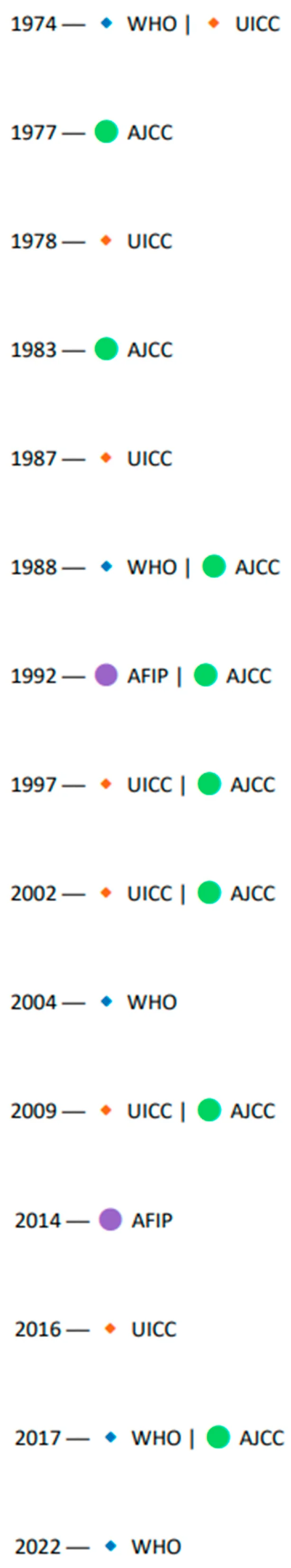
Chronological milestones derived from the combined annual timeline for thyroid cancer classifications.

Based on a single timeline for each TC classification (Figure 1), we combined classifications from the same year into a single timeline and therefore, we identified the following 15 milestones: 1974 (UICC), 1977 (AJCC), 1978 (UICC), 1983 (AJCC), 1987 (UICC), 1988 (WHO and AJCC), 1992 (AFIP and AJCC), 1997 (UICC and AJCC), 2002 (UICC and AJCC), 2004 (WHO), 2009 (UICC and AJCC), 2014 (AFIP), 2016 (UICC), 2017 (WHO and AJCC), and 2022 (WHO) (Figure 2).

### 3.2. Temporal Dynamics for PTC and FTC Historical Time Series

#### 3.2.1. PTC vs. FTC Series

In 1975, the AIR (per 100,000 persons) was 3.6 for PTC [95% CI: 3.42–3.78] but 0.7 for FTC [95% CI: 0.61–0.79], representing a 5-fold baseline difference (Table 2). In 2023, the AIR of PTC reached 12.8 [95% CI: 12.45–13.15] (representing a marked expansion compared to 1975), whereas that of FTC changed to 1.2 [95% CI: 1.08–1.32] (Table 2). In percentage terms, the AIR of PTC expanded by 256% compared to 71% for FTC (Table 2). Consequently, by 2023, the aggregate gap between the AIR of PTC and the AIR of FTC widened compared to the 1975 baseline parameters (10.67 vs. 5.14).

#### 3.2.2. PTC and FTC Series Side-by-Side

**Figure 3:**
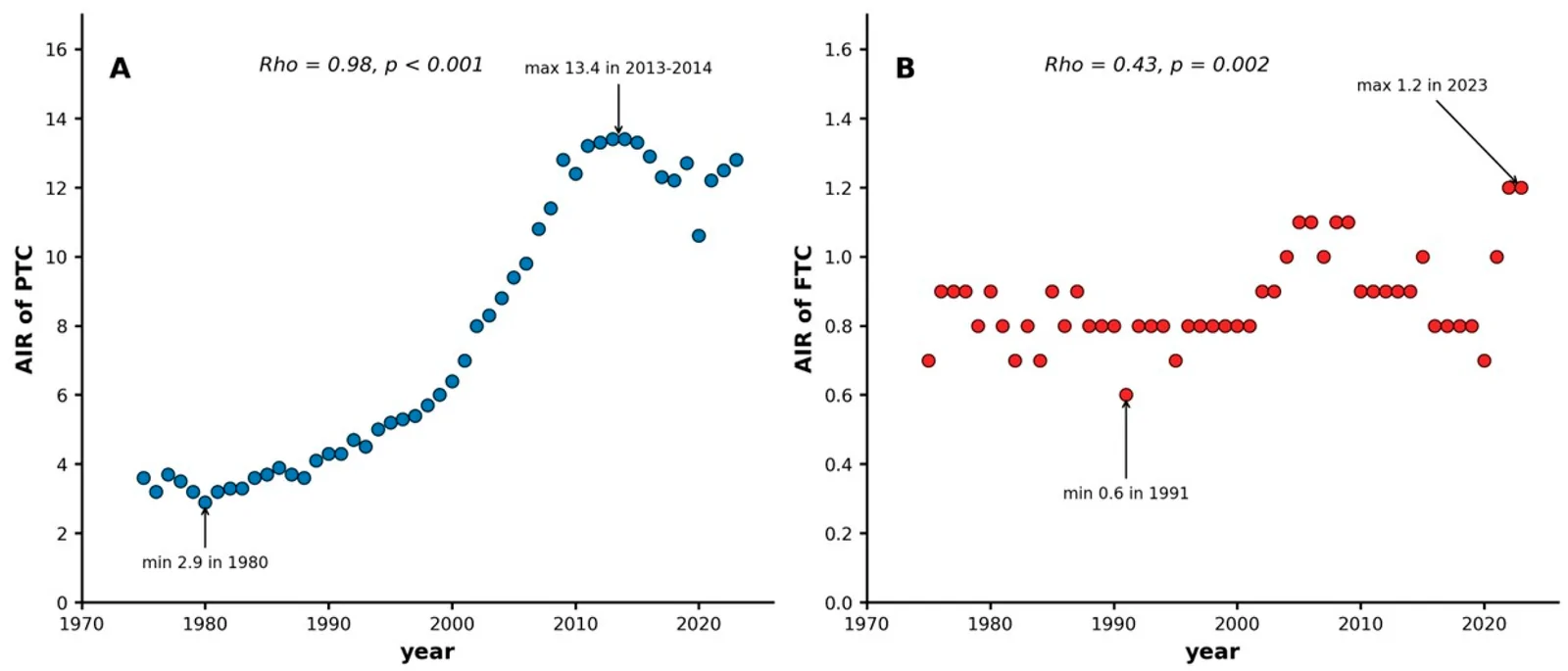
Chronological incidence trends for papillary and follicular thyroid cancers. Footnotes: (**A**) Historical trend of the AIR for papillary thyroid cancer, highlighting the absolute minimum in 1980 (2.9) and the peak plateau maximum in 2013–2014 (13.4); a strong positive correlation with time was observed (Rho = 0.98, *p* < 0.001). (**B**) Fluctuation boundaries and stability analysis of the AIR for follicular thyroid cancer, indicating the historical minimum in 1988–1991 triennium (0.6) and the maximum value achieved in 2023 (1.2), demonstrating a moderate but significant temporal trend (Rho = 0.43, *p* = 0.002).

In the 49-year period spanning 1975 to 2023, the 49 annual data points of the PTC series showed a progressive upward trend. For PTC, the lowest historical AIR (2.9 per 100,000 population) was in 1980 (Table 3 and Figure 3). Starting from this node, the AIR of PTC expanded until it reached a maximum value of 13.4 in the 2013–2014 biennium (Table 3 and Figure 3). Subsequently, a transient downward fluctuation was recorded, as shown by an AIR of 10.6 in 2020 and 12.8 in 2023 (Figure 3), reflecting a nonlinear, non-continuous historical trajectory. Over the 49 years, the mean (M) ± SD of the PTC series was 7.60 ± 3.95 per 100,000, with a median (Mdn) of 6.00 (Table 3). The mean value being higher than the median points toward a right-skewed asymmetry, consistent with the higher levels recorded in recent decades (Table 3 and Figure 3). Finally, the relatively high SD and variance (15.647) reflect a significant dispersion around the mean value, which is consistent with the steep upward trajectory of the series (Table 3).

In the same 49-year period, the AIR for FTC fluctuated within a restricted range, with a baseline minimum of 0.6 per 100,000 (occurring in 1991) up to a maximum of 1.2 in 2023 (Table 3 and Figure 3). A stable phase at 1.0–1.1 per 100,000 occurred from 2004 through 2009, followed by a localized variation from 2020 (0.7 per 100,000) through 2023, with an AIR of 1.0 in 2021 and 0.9 in 2022 (Figure 3). Small differences in FTC incidence are not evaluated as clinical shifts unless formal parameters and uncertainty intervals support such a conclusion. Since the sample mean (0.86) and median (0.80) were close to each other, the visual symmetry of the distribution was evaluated via a formal Shapiro–Wilk normality test, which failed to reject the null hypothesis of normality (W = 0.958, *p* = 0.082). This inferential validation confirms that the FTC series is consistent with an underlying Gaussian framework, displaying a low standard deviation (±0.12) and a variance close to zero (0.014) (Table 3).

**Figure 4:**
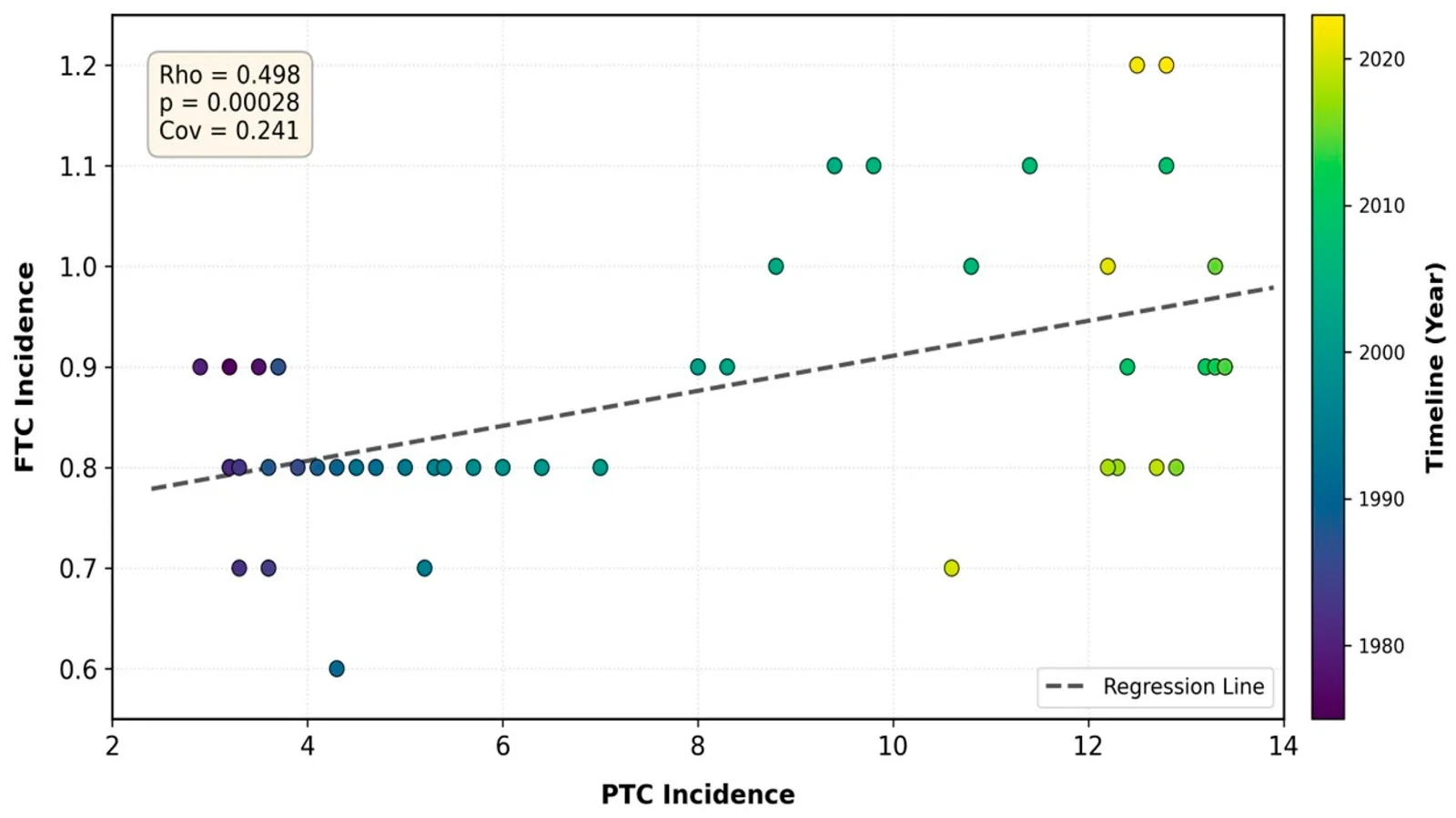
Correlation analysis and temporal evolution of papillary and follicular thyroid cancers incidences (1975–2023). Footnotes: The figure illustrates the moderate positive correlation between papillary thyroid cancer (PTC) and follicular thyroid cancer (FTC). Individual data points are color-coded according to the timeline progression, ranging from dark purple (1975) to bright yellow (2023). Statistical markers (Cov = 0.241, *r* = 0.498, Rho = 0.498, *p* = 0.00028) and the dashed linear regression line confirm a shared macro-epidemiological trajectory, although their annual fluctuations remain partially independent. Notably, the peak maximum for PTC (13.4) forms a stable plateau spanning the 2013–2014 period.

Association analyses were conducted to evaluate whether the two histotypes shared common epidemiological dynamics. While the raw co-movement parameters suggest a positive covariance (Cov = 0.241) and a moderate positive correlation (r = 0.498, *p* < 0.001) on the original timelines, these metrics are subject to temporal autocorrelation and strong, shared chronological trends. To control for potential spurious correlation, the time series were analyzed by taking first differences (ΔPTC*t* and ΔFTC*t*) to evaluate year-to-year changes. Crucially, the first-difference correlation coefficient drops to approximately *r*Δ= 0.110 and loses all statistical significance (*p* = 0.453). This indicates that there is little contemporaneous linear association in annual changes once common trends are removed. However, this lack of correlation describes the lack of temporal co-movement in the series and does not by itself establish causal or biological independence (Table 3 and Figure 4).

The numerical alignment between the raw Pearson correlation coefficient (r = 0.498) and the raw Spearman rank coefficient (ρ = 0.498) is consistent with the highly steady monotonic features shared by both historical series across the timeline. Since the FTC dataset is consistent with a stable Gaussian distribution and appears free from extreme outlying fluctuations, the linear coordinates tend to correspond closely with the ordinal rank assignments prior to filtering. This baseline overlap is compatible with a shared macro-historical trend, which supports the application of the subsequent first-difference analysis (*r*Δ = 0.110, *p* = 0.453) to better evaluate the potential epidemiological independence of the two histotypes.

Population data were retrieved from the SEER database and filtered utilizing the SEER Site Recode ICD-O-3/WHO 2008 variable, restricted specifically to the Thyroid site. The extracted dataset encompasses both sexes and includes the total aggregate population without distinctions by race to guarantee an unselected, population-level baseline representation. The Diagnostic Confirmation variable was restricted exclusively to Microscopically confirmed cases. PTC Histology Type (Aggregate Papillary Cohort): Encompasses ICD-O-3 morphology codes 8050, 8052, 8260, 8340 (Follicular Variant of PTC), 8341 (Papillary Microcarcinoma), 8342, 8343, and 8344. FTC Histology Type (Aggregate Follicular Cohort): Encompasses ICD-O-3 morphology codes 8290 (Oxyphilic adenocarcinoma/Hürthle cell carcinoma), 8330, 8331, 8332, and 8335. Abbreviations: AIR: Annual Incidence Rate; FTC: Follicular Thyroid Carcinoma; ICD-O-3: International Classification of Diseases for Oncology, 3rd Edition; PTC: Papillary Thyroid Carcinoma; SEER: Surveillance, Epidemiology, and End Results Program.

### 3.3. Endogenous Breakpoints in FTC and PTC Historical Time Series and Their Temporal Associations with TC Classifications

Three complementary statistical frameworks were deployed to map the structural breakpoints within the FTC and PTC historical timelines. As per protocol (see Materials and Methods), the analytical sequence initiated with an automated screening procedure, followed by the sequential implementation of the PSR-RS and MSIA models. To ensure maximum analytical robustness, this protocol was executed through two distinct, alternative approaches: data-driven and classification-driven.

By the first approach, the historical series were analyzed independently from any classification timelines. Crucially, to eliminate visual selection bias and guarantee algorithmic reproducibility, the candidate breakpoints did not emerge from visual data inspection; instead, they were endogenously isolated via an automated recursive Supremum Chow Test script in R software utilizing the “strucchange” package [49]. In a subsequent step, these endogenously identified structural breakpoints were chronologically aligned with the historical timelines of international classifications to evaluate potential temporal associations.

By the second approach, the candidate breakpoints were strictly fixed to match the exact publication years of each classification framework (WHO, UICC, AJCC, AFIP). Under both analytical approaches, the 1974 and 2022 classifications were omitted from the segmentation analysis, as they corresponded to the boundary years of the time series, thus reducing the minimum degrees of freedom required to compute the partitioned sub-period parameters.

#### 3.3.1. Approach 1. Chronology of the Endogenous Structural Breakpoints

**Figure 5:**
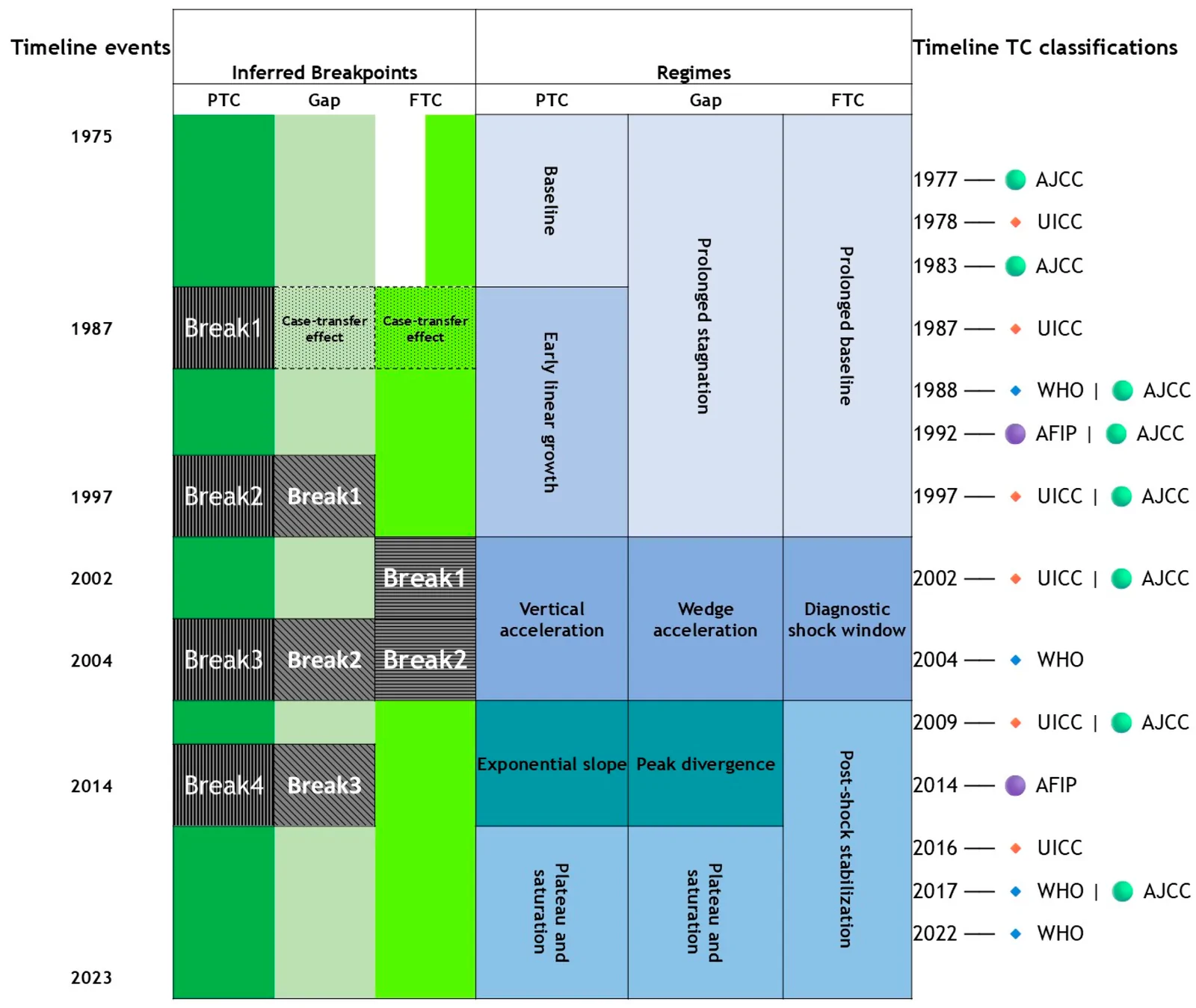
Natural breakpoints and evolutionary regimes for PTC, FTC, and absolute gap time series from 1975 to 2023. Footnotes: this synthetic chronological mapping aligns historical timeline events with classification updates for thyroid cancer (TC), demonstrating how successive international staging and histopathological classification updates display temporal associations with systematic reporting shifts and diagnostic criteria variations at a population level. The left panels delineate the inferred natural breakpoints, showcasing a consistent temporal synchronization between papillary thyroid cancer (PTC) and the absolute observational gap shifts, which is uniquely decoupled during the 1997 adjustments phase. Grounded in these classification updates, the central panel tracks the corresponding evolutionary regimes from 1975 to 2023, illustrating how successive diagnostic criteria trigger distinct regimes, including vertical accelerations, peak divergence, and late-stage post-shock stabilization. Finally, the right panel details the chronological evolution of TC classifications (AJCC, UICC, WHO, and AFIP), providing the direct regulatory and diagnostic context that shapes each independent epidemiological era.

Under the automated data-driven approach, the recursive scanning algorithm detected a total of nine natural structural breakpoints (Figure 5). Four endogenous breaks were identified within the PTC series, occurring in the years 1987, 1997, 2004, and 2014 (Figure 5). Two breakpoints were isolated in the FTC series, occurring in 2002 and 2004 (Figure 5). The remaining three structural nodes were captured within the aggregate gap timeline in 1997, 2004, and 2014 (Figure 5).

Chronologically, the first PTC structural break of late 1987 aligned with the publication axis of the 4th edition of the UICC staging classification (Figure 5). The two structural breaks isolated in 1997 corresponded to the publication year of the 5th editions of both the UICC and AJCC frameworks (Figure 5). The three simultaneous breakpoints of the year 2004 emerged across the individual PTC and FTC series as well as their cognate gap timeline; the 2004 node coincided with the publication axis of the 3rd edition of the WHO classification. The FTC breakpoint of 2002 was aligned with the publication year of the 6th editions of both the UICC and AJCC classifications (Figure 5). Finally, the two structural milestones of 2014 recorded in the PTC series and in the aggregate gap corresponded to the publication year of the 4th edition of the AFIP Atlas of Tumor Pathology.

To prevent statistical circularity, the candidate years isolated by the initial automated filter were subjected to independent inferential validation using subsequent controls. A breakpoint is formally verified only if it displays a statistically significant slope modification (Δ*β*) under the PSR-RS model or an active latent state transition probability within the MSIA framework. The convergence between the data-driven nodes of Approach 1 and the forced publication years of Approach 2 is highly consistent with a robust population-level epidemiological signal rather than an artifact of data selection.

#### 3.3.2. Phenotypes of the Natural Structural Breakpoints

By the joint PSR-RS and MSIA parameterization, the identified data-driven breaks did not exhibit the same statistical phenotype (Table 4). Rather, five distinct phenotypes (denominated A, B, C, D, and E) of structural breaks emerged across the timelines: three for the PTC series and two for the FTC series (Table 4). Phenotype A appeared in 1987 (PTC series) and 2014 (PTC series and aggregate diagnostic gap) (Table 4). This phenotype consisted of a progressive slope modification, typified by a trajectory transition through a significant progressive slope modification (Δβ) in the absence of a statistically detectable or significant immediate level shift (β_2_) under standard significance boundaries (Table 4). Under this simultaneous estimation, the 1987 PTC structural break (*F* = 24.31, *padj* < 0.01; Δ*β* = +0.2868, *p* = 0.002) acted as a significant trajectory turning point for the long-term papillary series, anchoring the permanent slope redirection of the subsequent regime.

Conversely, the 1997 structural breakpoint represented a distinct parameter configuration. Unlike the shifts associated with major histopathological updates, the 1997 milestone emerged as an endogenous transition primarily captured within the latent regime parameters. This break represents an operational transition point where the underlying time series adjusted without an abrupt level shift, aligning with the introduction of the 5th editions of the UICC and AJCC frameworks. The formal parameterization confirms that the 1997 axis reflects a steady continuation of the existing acceleration parameter, behaving as a statistical baseline adjustment within the population-level registry reporting framework.

Symmetrically, the FTC series experienced a localized fluctuation during the 1988–1991 period (Figure 3), though population-level data at this aggregate layer do not permit the evaluation of active, patient-level diagnostic migration. Both the 2014 PTC natural break (*F* = 72.15, padj < 0.01; Δβ = −0.3652, *p* = 0.035) and the aggregate diagnostic gap break (*F* = 59.30, padj < 0.01; Δβ = −0.35, *p* = 0.038), belonging to phenotype A, shared a similar structural parameter configuration to the 1987 trend adjustment (Table 4). However, the PSR-RS framework isolated a significant negative slope modification for the 2014 nodes, indicating a shift from the previous upward trajectory to a structural stabilization into a plateau (Figure 5), marking a distinct transition from the rapid growth phase.

The 1997 PTC structural break constituted phenotype B, which is restricted to the PTC series, representing a shared parameter configuration across both time-series frameworks (*F* = 79.52, padj < 0.001; β_2_ = +2.0996, *p* = 0.002; Δβ = +0.1771, *p* < 0.001; implicit intercept shift = +1.0940, *p* < 0.001) (Table 4).

Under the PSR-RS model, this structural node corresponded to a deterministic level adjustment and a slope modification (Table 4). Simultaneously, the probabilistic implicit intercept and slope shifts estimated under the MSIA model mirrored the parameters of the linear framework, validating these transitions within the latent parameters (Table 4).

Conversely, the 2002 FTC node, designated as phenotype C, yielded an adjusted p-value above the formal significance threshold (*p_adj_* = 0.0575) following the application of the Benjamini–Hochberg multiplicity correction. Consequently, this axis is interpreted as a transitory operational fluctuation rather than a validated structural rupture, displaying a localized level variation without generating a permanent trend acceleration (Table 4).

In 2004, both the PTC series and the aggregate diagnostic gap timelines exhibited phenotype D (Table 4), characterized by pure deterministic level adjustments (*F* = 158.46, padj < 0.001 and *F* = 142.18, padj < 0.001, respectively). Symmetrically, the 2004 FTC node represented phenotype E, exhibiting a moderately significant vertical adjustment following multiplicity correction (*F* = 4.85, padj= 0.0200) (Table 4). The 2004 breakpoints associated with phenotype D and E express distinct statistical features, as phenotype D lacks long-term slope modifications (Δβ non-significant) and MSIA transition dynamics, operating as a localized operational adjustment within the population-level timeline. The 2004 parameters represent a definitive structural milestone for PTC, characterized by a systematic vertical jump (*β*2 = +4.1500, padj < 0.01) that aligns temporally with the updated criteria of the WHO 3rd Edition (Table 4). Because the FTC series remained stable or fluctuated slightly above the threshold of 1.0 per 100,000 population during this period, this post-2004 vertical expansion occurred independently of any aggregate case-redistribution effects (Figure 3).

#### 3.3.3. Regimes and Natural Breakpoints

The identified data-driven natural breaks partition the historical timelines into distinct epidemiological phases. For the PTC series, four breakpoints subdivide the continuous timeline into five sequential regimes. Regime 1 (initial baseline) spanned the 1975–1986 period and was characterized by a stable baseline incidence, with the AIR starting at 3.6 and ending at 3.7 per 100,000 (Figure 3 and Figure 5). Regime 1 was followed, in the years 1987–1996, by Regime 2 (early linear trend modification, with the AIR starting at 3.7 and ending at 5.4) (Figure 3 and Figure 5). This segment was anchored by the 1987 structural milestone, which marks the departure from the initial baseline status toward a moderate expansion, aligning chronologically with the implementation of the 1987 UICC 4th Edition staging (Figure 5).

Regime 2 corresponded to a phase of international standardization of criteria, which separated PTC from similar clinical entities based on histological classifications. Regime 3 (accelerated phase) spanned the 1997–2003 interval, with the AIR starting at 5.4 and ending at 8.8 per 100,000 (Figure 3 and Figure 5), initialized around the 1997 structural axis (Figure 5). This phase represented a clear expansion of baseline levels and annual growth rates. Regime 4 (rapid linear acceleration) encompassed the 2004–2013 timeframe (Figure 5), with the AIR expanding from 8.8 to 13.4 (Figure 3). This segment aligns with the standardized reporting criteria introduced around the 2004 WHO 3rd Edition, capturing a period of significant trajectory momentum (Figure 3).

Finally, Regime 5 (plateau phase) encompassed the 2014–2023 timeframe (Figure 5), with the AIR moving from 13.4 to 12.8 (Figure 3). The 2014 turning point marks the transition into this structural plateau, which is highly consistent with a synergistic cooperation between updated classification guidelines and contemporary technological advancements in clinical detection [83]. This modern era of diagnostic stabilization effectively modulated the previous upward momentum into a stable plateau, pointing toward a general trend stabilization.

This final segment included the modern era of diagnostic stabilization, successfully transforming the previous upward momentum into a stable plateau. Indeed, it marked a baseline shift toward a structural deceleration in the population-level behavior of PTC. Regime 5 is consistent with the general trend stabilization observed following the international implementation of preventive de-escalation strategies. The onset of Regime 5 in 2014 incorporates the historical transition toward more conservative guidelines, such as the subsequent reclassification of non-invasive encapsulated follicular lesions into NIFTP and the global adoption of the 2015 ATA guidelines [17,84]. By shifting clinical and pathological diagnostic thresholds for indolent or sub-centimeter lesions, these collective de-escalation strategies are consistent with the immediate saturation effect that stabilized the population-level trajectory. The 2014 turning point marks the transition into this structural plateau, which is highly consistent with a synergistic cooperation between updated classification guidelines and contemporary technological advancements in clinical detection.

In comparison with the PTC historical series, the identification of only two natural nodes (the 2002 transitory fluctuation and the 2004 moderately significant break) segment the FTC timeline into three distinct regimes (Figure 5). Regime 1 (prolonged baseline) occurred between 1975 and 2001, representing an extended phase of epidemiological stability where the AIR fluctuated marginally within a highly compressed range, remaining close to a mean value of 0.8 per 100,000 population. This extensive initial phase represented nearly three decades of clinical stability, during which the FTC series showed little modification during the early histopathological and staging updates that were simultaneously altering the PTC series (Figure 3 and Figure 5).

Regime 2 (transitory shifting phase) encompassed the brief 2002–2003 interval (Figure 5), with the AIR shifting from 0.9 to 1.0 per 100,000 (Figure 3). Although the nominal increase was limited (+1.1), this segment was initialized around the 2002 data node, which—following the application of the Benjamini–Hochberg multiplicity correction (p_adj = 0.0575) is characterized as a temporary operational fluctuation rather than a permanent structural rupture. During this short phase, the FTC timeline registered a localized level variation, aligning temporally with the introduction of the 6th editions of the UICC and AJCC frameworks, without establishing a long-term trend acceleration, acting as a temporary disruption to the baseline before the subsequent 2004 classifications took effect.

Regime 3 (modern registry stabilization) spanned the 2004–2023 timeframe (Figure 5), with the AIR starting at 1.0 and ending at 1.2 [95% CI: 1.08–1.32] (Figure 3). Initiated by the 2004 milestone, which exhibited a moderately significant vertical adjustment (*β*_2_ = +0.2106, *padj* = 0.0200) accompanied by a localized slope modification (Δ*β* = −0.0104, *p* = 0.036), consistent with its assignment to phenotype E under standard significance boundaries (Table 5), this long-term final segment marked a definitive trend containment. While this represents a larger nominal increase (+0.2), the annualized growth rate slowed down, spreading the variation over nearly two decades. The FTC series permanently detached from its historical minimum (AIR of 0.6 in 1991) and entered into a stable, slightly fluctuating plateau (Figure 3).

When juxtaposing the two malignancies, the independent regime generation revealed a profound epidemiological asymmetry (Figure 5). While PTC required a highly fragmented, 5-regime evolutionary path, FTC was characterized by an enduring structural inertia. Therefore, the PTC series exhibited a higher susceptibility to responding to systematic diagnostic shifts since 1987, whereas the timelines of the two malignancies converged exclusively during the 2002–2004 window.

The structural regimes governing the absolute gap did not mirror the 5 regimes of the PTC series, collapsing instead into four distinct behavioral segments (Figure 5). This divergence is highly consistent with the asymmetrical variance and distinct trend parameters of the individual histotypes. While the PTC series incurred its initial trend break in 1987 (entering into an early linear growth phase), the aggregate gap parameters dampened this shift due to the contemporary stability recorded in the FTC series. Consequently, the gap framework bypasses the 1987 milestone, remaining bound to a singular prolonged stagnation regime (1975–1997). It is only after the 1997 axis that the gap aligned in close chronological synchronization with the structural phases of PTC (Verticalization, Peak Divergence, and Saturation Plateau in Figure 5), demonstrating that the long-standing expansion of the diagnostic wedge became a prominent population-level pattern following the late 1990s clinical paradigm shifts.

#### 3.3.4. Approach 2. Generated Breakpoints Through TC Timeline Classifications

Under the classification-driven (top-down) approach, the recursive framework evaluated the 13 fixed candidate years corresponding to historical classification releases (Table 5). Within the PTC series, the Chow filter identified temporal associations across these milestones, whereas in the FTC series, statistically significant adjustments were restricted to the 2002, 2004, and 2009 axes (Table 5). Regarding the aggregate diagnostic gap, significant parameters aligned with the dominant PTC timeline, with the exception of the 1977 and 1978 milestones, which displayed no significant variations (Table 5). Following the application of the Benjamini–Hochberg multiplicity correction across all 39 tests, these parameters must be interpreted with statistical caution, as marginal raw values inflation is effectively controlled.

A comparison between the trends estimated via the data-driven framework and the alternative top-down approach reveals distinct numerical behaviors in the piecewise regressions (Table 4 and Table 5).

With the exception of the 2004 node, the PSR-RS model under the top-down approach yielded similar coefficients for both β_2_ and Δβ across several forced milestones (Table 4 and Table 5). This convergence represents a mathematical consequence of the top-down constraint: since a uniform set of fixed institutional dates was simultaneously introduced into the equations, and because the absolute variance of the FTC series was highly compressed (AIR ranging from ~0.7 to 1.2), the geometric subtraction defining the gap shifted the curve downward while preserving its overall trajectory. Consequently, the forced piecewise segmentation of the aggregate gap closely mirrors the adjustments of the dominant PTC timeline.

Conversely, under the data-driven approach, this symmetry breaks down for the 1997, 2002, and 2014 milestones, which display divergent numerical values (Table 4 and Table 5). This variation is a known property of piecewise regressions, where individual parameter estimations depend entirely on the specific combination and total number of breakpoints active within the model matrix. In the data-driven approach, the year 2002 is endogenously isolated as a natural breakpoint exclusively for the FTC series (Table 4 and Figure 5). Crucially, following multiplicity adjustment, the 2002 FTC parameter falls outside the formal significance boundary (*padj* = 0.0575), characterizing this axis as a transitory operational fluctuation rather than a validated structural rupture.

For the PTC series, the model estimates its piecewise trajectories based on a 4-breakpoint configuration (1987, 1997, 2004, and 2014) (Table 4 and Figure 5). Within the aggregate gap timeline, however, the 1987 breakpoint is absent due to the asymmetrical variance and parallel stability of the follicular baseline, which mathematically cushioned the absolute gap calculation without requiring a patient-level case-redistribution hypothesis. This variation forces the gap model to operate on a 3-breakpoint configuration (1997, 2004, and 2014) (Table 4 and Figure 5). By altering the structural breaks anchoring the regression lines, the mathematical optimization process calculated distinct localized slope and intercept parameters for 1997 and 2014. Therefore, the numerical variation observed between the two protocols does not represent a statistical inconsistency; rather, it reflects the independent, data-driven nature of the first approach, which isolates the geometric evolution of the population-level divergence without artificial constraints.

When analyzing these breakpoints by approach 2, the parameters of PSR-RS model reveal a powerful statistical phenomenon. Within the PTC series, the breakpoints were associated with highly significant vertical jumps (β_2_) in years 1997, 2002, 2004, and 2009 (Table 5). Notably, the 1997 and 2004 breakpoints corresponded to the natural breakpoints identified through approach 1 (Table 4 and Table 5).

Conversely, the significance of β_2_ in 2002 (UICC/AJCC 6th edition) and 2009 (UICC/AJCC 7th edition) represented a localized mathematical reflection of the ongoing exponential momentum rather than independent evolutionary landmarks. This statistical behavior occurs because the post-1997 era was a phase of rapid linear acceleration (Figure 5). Therefore, by forcing fixed calendar milestones into periods immediately following a major structural trajectory change, the regression model registers pronounced numerical variations, generating localized adjustments in 2002 (β_2_ = +3.7641, *p* < 0.001) and in 2009 (β_2_ = +3.7199, *p* < 0.001) (Table 5).

**Figure 6:**
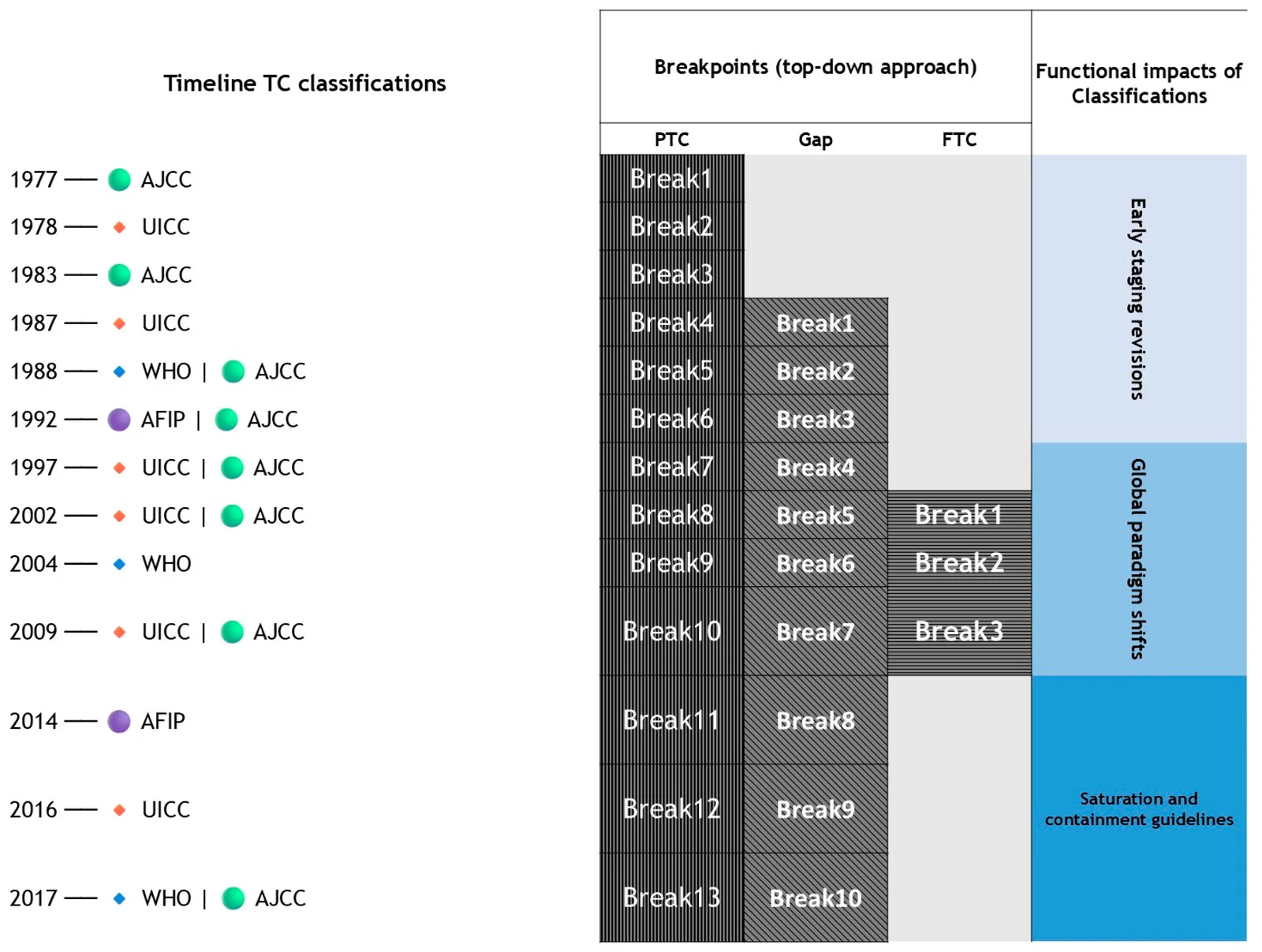
Classification-driven timeline analysis and functional impact eras for thyroid cancer (1975–2023). Footnotes: this classification-driven timeline aligns major historical staging updates with their specific regulatory outcomes, demonstrating how international consensus guidelines directly shape thyroid cancer epidemiological eras. The left panel details the chronological evolution of TC classifications across the AJCC, UICC, WHO, and AFIP staging systems from 1977 to 2017. Grounded in these updates, the central panel illustrates the cascading sequence of top-down breakpoints across PTC (Break1–13), FTC (Break1–3), and the absolute gap (Break1–10), capturing the exact structural segments triggered by each date. Finally, the right panel groups these events into three distinct phases of functional impact, tracking the operational transition of the system from early long-term upward catalysts (1977–1992) and highly synchronized global paradigm shifts (1997–2009) to modern saturation and containment strategies (2014–2017) designed to curb overdiagnosis.

Under this classification-driven approach, the chronological trends can be systematically categorized into three distinct phases of reporting impact: “early staging and classification alignments,” “central reporting adjustments,” and “stabilization and trend containment” (Figure 6). The “early staging and classification alignments” appeared in the 1977–1992 timeframe (AJCC 1977/1983, UICC 1978/1987, WHO 1988, and AFIP 1992) (Figure 6). These updates acted as milestones primarily for the PTC series because they exerted an impact exclusively on the slope parameter (Δβ) of PTC, leaving the vertical level adjustment (β_2_) non-significant (Table 5). This demonstrates that early diagnostic updates did not induce abrupt, localized level changes, but are consistent with a gradual, long-term upward trend.

In contrast, the “central reporting adjustments” occurred between 1997 and 2009 (Figure 6). This timeframe corresponded to an era when classification updates aligned with parameters across both histotypes, showing a co-activation of vertical adjustments (β_2_) and slope modifications (Δβ) (Table 5). Within the FTC series, in addition to the 2004 structural breakpoint, the 2002 fixed milestone displayed a level adjustment under raw estimation (β_2_ = +0.2337, *p* < 0.001), which must be interpreted conservatively as a localized registry variation following multiple-testing controls.

The final phase tracked on the timeline, “stabilization and trend containment,” ranged between 2014 and 2017, revealing a significant slowing down of the trends (Figure 6). The 2014 AFIP Atlas milestone corresponds to a significant negative slope modification (Δβ = −0.35) in the PTC series, which is highly consistent with a synergistic cooperation between updated conservative guidelines and contemporary technological advancements in clinical detection. This convergence successfully stabilized the previous upward momentum, flattening the population-level data into a structural plateau that rendered subsequent updates (UICC 2016, WHO/AJCC 2017) statistically non-significant within this timeframe (Figure 6).

## 4. Discussion

The historical trajectories of FTC and PTC reveal divergent temporal patterns in population-level statistics. Both series begin at relatively low rates but follow distinct paths, with the annual incidence rates (AIR) showing a widening aggregate diagnostic gap between the two malignancies. PTC remains consistently more frequent than FTC from 1975 through 2023. The aggregate gap increased from 2.9 per 100,000 population in 1975 (PTC AIR of 3.6 minus FTC AIR of 0.7) to 11.6 in 2014 (12.8 minus 1.2) and remained at a stable plateau thereafter (gap = 11.6 in both 2022 and 2023). While FTC incidence remained relatively stable with limited fluctuations, PTC incidence increased until approximately 2013–2015 period, and subsequently stabilized.

The integration of three complementary statistical frameworks (Supremum Chow Test, PSR-RS, and MSIA), implemented through a two-pronged approach (data-driven versus classification-driven), allows us to evaluate how international classification updates align with the epidemiological trends of FTC and PTC over nearly five decades (1975–2023). Rather than acting as neutral diagnostic frameworks, successive revisions by the WHO, UICC, AJCC, and AFIP show prominent temporal associations with structural changes, shifts in incidence trends, and the widening divergence (aggregate gap) between the two malignancies. A comparison of the aggregate gap across both approaches suggests that changes in histopathological classifications are highly consistent with systematic reporting shifts in epidemiological data.

Under the data-driven Approach 1, 1987 was identified as a significant structural breakpoint in the PTC curve, marking a progressive change in slope (Δβ = +0.2868). Notably, this year was not identified as an independent natural breakpoint in the aggregate gap series under Approach 1. This variation is consistent with the timing of the 1987 UICC fourth-edition staging framework and the subsequent 1988 WHO classification [54,66]. The 1988 guidelines standardized PTC classification based on ground-glass nuclear features, moving beyond follicular architecture alone [54]. At the population level, this transition coincided with stabilization of the follicular baseline alongside the early expansion of the papillary series. These asymmetrical patterns were accompanied by a continuous aggregate gap trajectory, with no data-driven structural break detected until the central reporting adjustments of 1997.

Under the data-driven Approach 1, 2002 was identified endogenously as a natural breakpoint only in the FTC series, representing a localized level shift (β_2_ = +0.2337). However, after the application of the Benjamini–Hochberg correction for multiple testing, this parameter did not meet the conventional threshold for statistical significance (adjusted *p* = 0.0575), characterizing this axis as a transitory operational fluctuation within the registry rather than a permanent structural rupture. This localized change was not identified as a breakpoint in the aggregate gap series under Approach 1; the FTC level shift was numerically smaller than the contemporaneous increase in the PTC series. However, under the top-down Approach 2, the fixed 2002 calendar milestone was associated with a localized adjustment in the aggregate gap. This statistical behavior is consistent with the introduction of the sixth editions of the UICC and AJCC frameworks, which aimed to refine the reporting of minimally invasive lesions, and was consistent with a localized registry adjustment without a sustained acceleration in the trend (Table 5) [61,69].

In contrast, the 2004 WHO third edition corresponds to a major structural milestone detectable across the series under both approaches. For the PTC series, it is associated with a systematic upward level shift (*β*2 = +4.1500, adjusted *p* < 0.01), with the AIR increasing from 8.8 to 12.8 by 2009. Notably, the FTC series did not show a corresponding decline during this decade; instead, it rose from its historical minimum and stabilized slightly above 1.0 per 100,000 population. This population-level behavior suggests that the post-2004 vertical expansion occurred independently of any aggregate case-redistribution effects. The 2004 WHO classification standardized the reporting criteria for papillary microcarcinomas [55]. By dedicating a specific section to these sub-centimeter lesions, the updated guidelines provided a standardized reporting framework that interacted with contemporary technological advancements in clinical detection, such as high-resolution ultrasonography and fine-needle biopsy, aligning with the maximum expansion of the diagnostic gap [84].

Both analytical approaches converge on 2014 milestone as a significant historical turning point, coinciding with the publication of the 4th edition of the AFIP Atlas of Tumor Pathology [52]. Characterized by a slope change (phenotype A), this milestone corresponds to significant negative slope changes in both the PTC series (Δβ = −0.3652) and the aggregate gap (Δβ = −0.35). This deceleration marks the beginning of the plateau phase (Regime 5). This structural stabilization is highly consistent with a synergistic cooperation between updated conservative guidelines and contemporary technological advancements in clinical detection. After decades of rising incidence in indolent lesions, the 2014 updates aligned with a broader international consensus toward trend containment. These guidelines raised diagnostic thresholds and preceded the formal reclassification of non-invasive encapsulated follicular lesions as NIFTP [52,85,86]. This broader de-escalation movement coincided with the stabilization of the previously rising PTC incidence trend.

Conventional segmented regressions have been deployed independently in TC epidemiology to track temporal accelerations in disease incidence [6,87]. Moving beyond fixed-trend models, recent healthcare research has coupled the Chow test with MSIA models to distinguish whether system transitions align with exogenous legislative changes at fixed dates or correspond to latent shifts within the population [88]. Additionally, in environmental frameworks, the combination of the PSR-RS model with the MSIA framework has been utilized as a cross-validation strategy to capture complex, non-linear trajectories [89].

Our analytical design complements these frameworks by moving from single-disease analysis to direct modeling of the cognate gap between PTC and FTC incidence using a multistage protocol. This approach connects deterministic level adjustments with probabilistic latent state transitions to evaluate long-term epidemiological trends. The aggregate gap widened from a 5-fold baseline difference in 1975 to a 10.6-fold difference by 2014. However, the 2014 trend containment corresponds to a statistical limit to this divergence. By establishing a structural plateau, this mechanism stabilized the population-level landscape. Consequently, subsequent staging updates (UICC 2016, WHO/AJCC 2017) became statistically non-significant within this timeframe.

Recent studies, such as Chen et al., indicate that clinical guidelines are temporally associated with a slowing growth in TC incidence [90]. Although Chen et al. analyzed trends from 1975 onward by evaluating each disease curve separately, they did not investigate the aggregate gap over time. In contrast, our multi-stage protocol focuses directly on the gap parameters, successfully accounting for the variance asymmetry between the two histotypes without requiring patient-level adjustments phase assumptions. The data indicate that the late 1980s updates did not widen the aggregate gap immediately, but corresponded to an expansion of the papillary series alongside a stable follicular baseline, which reached a historical level of 0.6 AIR between 1988 and 1991. While current literature focuses heavily on overdiagnosis as a uniform trend, our analytical protocol suggests that changing reporting criteria and classification updates act as prominent, quantifiable factors on registry data.

This investigation evaluates how successive classifications and diagnostic updates align with the modern epidemiological landscape of TC. By organizing the fixed, top-down breakpoints into three distinct phases, the protocol highlights a clear chronological shift in reporting patterns. The initial phase (1977–1992) is consistent with a gradual, long-term upward trend in PTC incidence without immediate vertical level shifts, reflecting an early stage of reporting expansion. This was followed by the central phase (1997–2009), which marks a period of synchronized adjustments, showing concurrent structural transitions and vertical level shifts across the series. Finally, the stabilization phase (2014–2017) captures the implementation of modern conservative guidelines. Overall, these findings suggest that the historical changes in TC trends are not merely random fluctuations, but show prominent temporal associations with the successive updates to international staging and classification standards.

## 5. Limitations of the Study

The findings of this study should be interpreted with several methodological and structural limitations in mind. First, this research relies on an observational, and uncontrolled design restricted to a single population-based database (US-SEER). Consequently, the identified temporal associations between statistical checkpoints and the publication dates of major classifications are consistent with—but do not establish—direct causal relationships. The design does not incorporate a separate comparison population, a negative control outcome, a counterfactual scenario, or a direct measurement of individual clinician and pathologist adherence to updated criteria.

Second, a primary challenge in breakpoint analysis involves treating the official publication dates of international classifications as precise proxies for the exact moment these frameworks began influencing clinical diagnosis and registry coding. In practice, a classification published in a particular year may not have been immediately or uniformly adopted by all pathologists, introducing a period of gradual consensus transition. To evaluate this fluid behavior within our time-series analysis, our statistical protocol deployed a multi-stage approach where the non-linear MSIA framework functions alongside segmented linear models (Chow test and PSR-RS). Because the MSIA framework computes unobserved transition probabilities driven by the data’s internal dynamics, it evaluates the continuous timeline without assuming immediate behavioral shifts, successfully accommodating gradual transitions in pathological habits.

Symmetrically, PSR-RS framework does not capture these early, faint consensus signals; instead, its strength lies in evaluating the consolidated, long-term trends once the guidelines have been widely assimilated by the medical community. In practice, the PSR-RS model is implemented through a multi-stage computational sequence using R software version 4.4.1 via the segmented package.

Third, a clear distinction must be made regarding how different institutional updates influence population-level registration. While histopathological classification handbooks (such as the WHO monographs) directly redefine microscopic diagnostic thresholds, staging systems (such as the UICC and AJCC manuals) do not rewrite histopathological definitions. Instead, staging frameworks exert an indirect impact on raw incidence counts. This indirect mechanism operates by altering clinical and surgical thresholds (such as size parameters or risk categories), which modifies surgical practices and the subsequent volume of specimens sent to laboratories. Furthermore, tumor registries follow specific coding manuals integrated with active staging systems; thus, staging revisions systematically modify reporting forms and data entry behaviors within central databases, resulting in registry reporting variations.

Fourth, a fundamental counter-validation involves the interaction between textbook criteria and the entry-into-force dates of regional registry coding rules, specifically ICD-O-2 (1992) and ICD-O-3 (2001) (Figure S1 in Supplementary Materials). Empirical testing confirms that these technical transitions do not correspond with the endogenous, data-driven checkpoints isolated within the gap timeline. For instance, fixing the model to the 1992 ICD-O-2 implementation yields non-significant results (F = 2.12, *p* = 0.1317), as the statistical trajectory shift occurred earlier, in late 1987 (F = 18.24, *p* < 0.001). This 1987 checkpoint captures the operational closure of the historical SEER 4-digit Extent of Disease framework (1983–1987) and the training transition toward the 10-digit system (EOD-88), aligning with the clinical reporting thresholds introduced by the 1987 UICC 4th Edition staging system well before manual distribution. Similarly, the 2001 ICD-O-3 implementation timeline remains statistically non-significant for a structural change, as the true trajectory redirection is endogenously isolated during the 2004 WHO milestone (F = 142.18, *p* < 0.001). This statistical mismatch indicates that while coding rules provide the structural categories for data assignment, the latent transitions are highly consistent with pathologists shifting their diagnostic thresholds in response to clinical guidelines rather than simple recoding artifacts.

Fifth, the model must be contextualized alongside the continuous background influence of diagnostic technology, including the widespread diffusion of neck ultrasonography, ultrasound-guided fine-needle aspiration (FNA), and cross-sectional incidental detection. This technological expansion acts as a continuous background inflationary force that creates a vast pool of identified subclinical lesions. However, because our statistical protocol is designed to evaluate directional modifications in the long-term temporal trend rather than raw absolute volumes, it untangles this ongoing background pressure from the discrete interventions of international classifications. Within this framework, technology provides the baseline availability of detected nodules, while international consensus updates function as trajectory catalysts that redefine the boundaries of malignancy, guiding how those existing lesions are cataloged. This co-operative interaction is supported by the 2014 containment plateau: the clinical availability of ultrasound and FNA did not decline after 2014, yet the trajectory experienced a significant negative slope change (Δβ = −0.35), consistent with the introduction of updated conservative thresholds that successfully counterbalanced the continuous technological pressure.

Sixth, the model must account for the localized clinical disruptions and broader clinical screening recommendations that characterize the modern plateau phase (Regime 5). The sharp, temporary drop in incidence rates observed in the year 2020 (10.6 for PTC and 0.7 for FTC per 100,000 population) represents a transient diagnostic interruption associated with the COVID-19 pandemic. The temporary suspension of routine screenings, clinical access, and elective surgical procedures induced a short-term reduction in database entries, behaving mathematically as an operational outlier within an already established plateau, as verified by the subsequent recovery in 2021–2023 back to the pre-pandemic parameters (~12.8 for PTC and ~1.2 for FTC). Symmetrically, broader screening adjustments and clinical recommendations—such as the 2017 United States Preventive Services Task Force (USPSTF) statement against routine screening in asymptomatic adults, alongside general changes in screening intensity and access to care—represent critical context for contemporary endocrine oncology trends. Testing the 2017 recommendation as a fixed milestone yields statistically non-significant results for a structural trend modification under our analytical protocol. This outcome demonstrates that the trajectory flattening and the transition into the modern plateau had been endogenously established earlier, specifically at the 2014 milestone (Δβ = −0.35, *p* < 0.05). Consequently, subsequent clinical statements like the 2017 USPSTF recommendations did not initiate a new structural redirection; instead, they functioned as reinforcement tools that validated and consolidated a conservative clinical movement that was already fully operational within the timeline following the histopathological classification shifts.

Finally, a major structural boundary is imposed by the aggregate architecture of historical registry data. Because central cancer registries group sub-morphologies under broad histological ICD-O-3 codes over the 49-year continuous timeline, individual variant-level trends cannot be definitively untangled. Consequently, the identified parameters must be interpreted strictly as descriptive, aggregate shifts modulated by a potential combination of unmeasured epidemiological factors, including variations in screening intensity, healthcare access, changing registry coding rules, and localized guidelines adoption.

## 6. Conclusions

This study implements an advanced multi-stage validation protocol to evaluate the long-standing epidemiological divergence observed between FTC and PTC from 1975 to 2023. By combining deterministic segmentation with probabilistic modeling across two distinct analytical pathways, the framework evaluates the structural properties of these continuous time series, establishing key phases of trend adjustments. The population-level data demonstrate a persistent diagnostic gap between the two pathologies across the 49-year timeframe, with PTC consistently remaining more frequent than FTC. However, the absolute size of the aggregate gap widened, passing from 2.9 cases per 100,000 population in 1975 (PTC = 3.6; FTC = 0.7) to 11.6 cases per 100,000 population in 2014 (PTC = 12.8; FTC = 1.2), maintaining a stable plateau thereafter through 2023, while the baseline ratio expanded from a 5-fold to a 10.6-fold difference.

The 1987 data-driven milestone identifies a distinct structural transition in population trends, aligning with the chronological introduction of updated staging and histopathological criteria. Rather than establishing a patient-level case migration, the late 1980s updates coincided with an expansion of the papillary series alongside a highly stable follicular baseline, which reached a historical minimum of 0.6 AIR between 1988 and 1991. This asymmetrical variance mathematically cushioned the absolute gap calculation, explaining why the aggregate gap trajectory maintained continuity without a data-driven break until the central reporting adjustments of 1997.

The 2004 WHO 3rd Edition milestone corresponds to a prominent structural alignment across the models. By standardizing the reporting criteria for sub-centimeter lesions, these updated guidelines provided a formal framework that interacted with contemporary technological advancements in clinical detection, associated with a significant vertical adjustment in the PTC curve that expanded the aggregate gap toward its maximum.

Both analytical approaches converge on the 2014 milestone as a significant historical turning point, marking a transition toward trend containment and diagnostic stabilization. This milestone corresponds to a significant negative slope modification, which is highly consistent with a synergistic cooperation between updated conservative classification guidelines and contemporary technological advancements in clinical detection. By introducing more conservative diagnostic thresholds for indolent or non-invasive lesions, this collective de-escalation movement successfully stabilized the previous upward momentum, flattening the population-level data into a structural plateau and rendering subsequent updates statistically non-significant within this timeframe.

In summary, within the boundaries of an ecological observational design, the progressive divergence observed between PTC and FTC trends may not exclusively reflect an autonomous biological acceleration. Instead, these population-level variations are compatible with descriptive patterns of systematic reporting shifts and diagnostic criteria modifications introduced by successive international staging and histopathological classifications.

## Figures and Tables

**Table 1:** TC classifications: from inception to progress.

Medical Organization	Classification	Edition	Publication Year	Series and Fascicle	Curated by	References	Average Years of Publications
AFIP/ARP	AFIP Atlas of tumor pathology	I	1953	First Series, Fascicle 14	Warren S and Meissner W	[33]	18.0
		II	1969	Second Series, Fascicle 4	Meissner W and Warren S	[50]	
		III	1992	Third Series, Fascicle 5	Rosai J, Carcangiu ML and DeLellis RA	[51]	
		IV	2014	Fourth Series, Fascicle 21	Rosai J, DeLellis RA, Carcangiu ML, Frable WJ et al.	[52]	
		V	2025	Fifth Series, Fascicle 23	Ghossein R, Xu B, Barletta JA and Tallini G	[53]	
WHO	Histological classification of thyroid tumours	I	1974	1st Edn, No. 11	Hedinger C, Williams ED and Sobin LH	[34]	12.0
		II	1988	2nd Edn, No. 11	Hedinger C, Williams ED and Sobin LH	[54]	
		III	2004	3rd Edn, No. 8	DeLellis RA, Lloyd RV, Heitz PU, Eng C	[55]	
		IV	2017	4th Edn, No. 10	Lloyd RV, Osamura RY, Kloppel G, Rosai J	[35]	
		V	2022	5th Edn, No. 8	WHO classification of tumours editorial board	[56]	
UICC	TNM classification of malignant tumor	I	1968	1st Edn	Denoix P and Harmer MH, Copeland MM, Costachel O et al.	[36]	7.1
		II	1974	2nd Edn	Harmer MH	[57]	
		III	1978	3rd Edn	Harmer MH	[58]	
		IV	1987 ^	4th Edn	Hermanek P, Sobin LH	[59]	
		V	1997	5th Edn	Sobin LH, Wittekind Ch	[60]	
		VI	2002	6th Edn	Sobin LH, Wittekind Ch	[61]	
		VII	2009	7th Edn	Sobin LH, Gospodarowicz MK, Wittekind Ch	[62]	
		VIII	2016	8th Edn	Brierley JD, Gospodarowicz MK, Wittekind Ch	[63]	
		IX	2025	9th Edn	Brierley JD, Giuliani M, O’Sullivan B, Rous B et al.	[64]	
AJC/AJCC	The Manual for Staging of Cancer	I	1977	1st Edn	Beahrs OH, Carr DT, Rubin P	[37]	7.1
		II	1983	2nd Edn	Beahrs OH, Meyers MH	[65]	
		III	1988 ^	3rd Edn	Beahrs OH, Henson DE, Hutter RVP, Meyers MH	[66]	
		IV	1992	4th Edn	Beahrs OH, Henson DE, Hutter RVP, Kennedy BJ	[67]	
		V	1997	5th Edn	Fleming ID, Cooper JS, Henson DE	[68]	
		VI	2002	6th Edn	Greene FL, Page DL, Fleming ID, Fritz AG et al.	[69]	
		VII	2009	7th Edn	Edge SB, Byrd DR, Compton CC, Fritz AG et al.	[70]	
		VIII	2017	8th Edn	Amin MB, Edge SB, Greene FL, Byrd DR et al.	[71]	

Footnotes. ^ Although the formal agreement to unify criteria between the two scientific bodies was reached internationally in 1987, their respective editorial manuals followed slightly staggered publication schedules. The European counterpart, the UICC (TNM Classification of Malignant Tumours, 4th Edition), was officially published in 1987. Meanwhile, the American AJCC manual edited by Beahrs and colleagues (Manual for Staging of Cancer, 3rd Edition, published by J.B. Lippincott) hit medical bookstores in early 1988.

**Table 2:** Primary metrics for trends analysis.

Histotypes	AIR Value		AIR Value Changes		
	1975 [95% CI]	2023 [95% CI]	Absolute Change (AC) AIR2023-AIR1975 (pp)	Percentage Change (PC) AIR2023-AIR1975/AIR1975 × 100 (%)	Incidence Rate Ratio (RR) AIR2023/AIR1975 (%)
PTC	3.6 [3.42–3.78]	12.8 [12.45–13.15]	9.20	255.6	3.56
FTC	0.7 [0.61–0.79]	1.2 [1.08–1.32]	0.50	71.4	1.71
Absolute Gap (AG) PTC-FTC (pp)	2.9	11.6	8.7	300	4
Gap Ratio (GR) PTC/FTC (%)	5.14	10.67	5.53	107.58	2.07

Footnotes: Abbreviations. AIR = Annual Incidence Rate, PTC = Papillary Thyroid Cancer, FTC = Follicular Thyroid Cancer.

**Table 3:** Descriptive and inferential statistics.

Histotypes	Number of AIR (N)	Minimum Value (min)	Maximum Value (max)	Mean (M)	Median (Mdn)	Standard Deviation (n − 1) (SD ±)	Variance (n − 1) (VAR)	Normality Test (Shapiro–Wilk W)	Spearman Coefficient (Rho)	Covariance (Cov) (Cov)	Raw Pearson Correlation Coefficient (r)	Raw Spearman Coefficient (Rho)	Detrended Pearson Correlation Coefficient (rΔ)
PTC	49	2.9	13.4	7.60	6	3.95	15.647	0.841 (*p* < 0.001)	0.98	0.241	0.498 (*p* < 0.001)	0.498 (*p* < 0.001)	0.110 (*p* = 0.453)
FTC	49	0.6	1.2	0.86	0.8	0.12	0.014	0.958 (*p* = 0.082)	0.43				

Footnotes: Abbreviations: AIR = Annual Incidence Rate; PTC = Papillary Thyroid Cancer; FTC = Follicular Thyroid Cancer; N = Number of observations; min = Minimum value; max = Maximum value; M = Mean; Mdn = Median; SD (±) = Standard Deviation; VAR = Variance; Rho = Spearman rank correlation coefficient; Cov = Covariance; *p* = *p*-value (Statistical significance); r = Raw Pearson correlation coefficient; *r*_Δ_ = Detrended Pearson correlation coefficient calculated on first-differenced series.

**Table 4:** Approach 1 Results—Data-Driven Breakpoints and Classification Alignment.

Natural Breakpoints	Target Series	Chow Test		Segmented Piecewise Parameterization (PSR-RS)				Markov-Switching Intercept and Slope-MSIA				Phenotypes	Aligned TC Classification Matching
		F	*p*	Main Dummy (β_2_)	*p*-Value	Slope Modification (Δβ)	Segmented *p*-Value	Implicit Intercept Shift	*p*-Value	Implicit Slope Shift	*p*-Value		
1987	PTC	24.31	<0.0001 ***	−0.3146	0.666 n.s.	+0.2868 **	0.002	n.a.	n.a.	n.a.	n.a.	A	1987—UICC
1997	PTC	79.52	<0.0001 ***	+2.0996 **	0.002	+0.1771 ***	<0.001	+1.0940 ***	<0.001	+0.3010 ***	<0.001	B	1997—UICC|AJCC
	PTC/FTC GAP	68.12	<0.001	+2.02 **	0.003	+0.18 ***	<0.001	n.a.	n.a.	n.a.	n.a.	E	
2002	FTC	3.51	0.0383 *	+0.2337 ***	<0.001	−0.0060	0.167 n.s.	+0.1292 **	0.002	−0.0080 *	0.041	C	2002—UICC|AJCC
2004	PTC	158.46	<0.0001 ***	+4.1500 ***	<0.001	−0.0736	0.088 n.s.	n.a.	n.a.	n.a.	n.a.	D	2004—WHO
	FTC	4.85	0.0123 *	+0.2106 **	0.001	−0.0104 *	0.036	n.a.	n.a.	n.a.	n.a.	E	
	PTC/FTC GAP	142.18	<0.001	+3.94 ***	<0.001	−0.06	0.115 n.s.	n.a.	n.a.	n.a.	n.a.	D	
2014	PTC	72.15	<0.0001 ***	+0.7759	0.451 n.s.	−0.3652 *	0.035	n.a.	n.a.	n.a.	n.a.	A	2014—AFIP
	PTC/FTC GAP	59.30	<0.001	+0.65	0.512 n.s.	−0.35 *	0.038	n.a.	n.a.	n.a.	n.a.	A	

Footnotes. Background colors are explicitly coded to reflect the cumulative frequency of each structural phenotype across the time series. Phenotype A: (Frequency = 3); Phenotype E: (Frequency = 2); Phenotype D: (Frequency = 2); Phenotype B: (Frequency = 1); Phenotype C: (Frequency = 1). Asterisks denote statistical significance scales (* *p* < 0.05, ** *p* < 0.01, *** *p* < 0.001; n.s. indicates not statistically significant; n.a. (not applicable) labels cells where latent hidden states do not experience active transition differentials.

**Table 5:** Approach 2 Results—Classification-driven (top-down).

Classification Year	Framework/ Source	Target Series	Chow Test		Segmented Piecewise Parameterization (PSR-RS)				Markov-Switching Intercept and Slope-MSIA			
			F	*p*	Main Dummy (β_2_)	*p*-Value	Slope Modification (Δβ)	Segmented *p*-Value	Implicit Intercept Shift	*p*-Value	Implicit Slope Shift	*p*-Value
1977	AJCC	PTC	4.25	0.0204 *	−2.1946	0.079 n.s.	+0.2275	0.802 n.s.	n.a.	n.a.	n.a.	n.a.
		FTC	1.08	0.3487 n.s.					n.a.	n.a.	n.a.	n.a.
		PTC/FTC gap	1.15	0.3251 n.s.	−2.1946	0.079 n.s.	+0.2275	0.802 n.s.	n.a.	n.a.	n.a.	n.a.
1978	UICC	PTC	5.31	0.0084 **	−2.0688	0.067 n.s.	+0.2631	0.637 n.s.	n.a.	n.a.	n.a.	n.a.
		FTC	1.05	0.3582 n.s.					n.a.	n.a.	n.a.	n.a.
		PTC/FTC gap	1.34	0.2721 n.s.	−2.0688	0.067 n.s.	+0.2631	0.637 n.s.	n.a.	n.a.	n.a.	n.a.
1983	AJCC	PTC	11.24	<0.0001 ***	−0.7721	0.351 n.s.	+0.3436 *	0.030	n.a.	n.a.	n.a.	n.a.
		FTC	1.48	0.2384 n.s.					n.a.	n.a.	n.a.	n.a.
		PTC/FTC gap	2.15	0.1284 n.s.	−0.7721	0.351 n.s.	+0.3436 *	0.030	n.a.	n.a.	n.a.	n.a.
1987	UICC	PTC	24.31	<0.0001 ***	−0.3146	0.666 n.s.	+0.2868 **	0.002	n.a.	n.a.	n.a.	n.a.
		FTC	1.84	0.1705 n.s.					n.a.	n.a.	n.a.	n.a.
		PTC/FTC gap	18.24	<0.001 ***	−0.3146	0.666 n.s.	+0.2868 **	0.002	n.a.	n.a.	n.a.	n.a.
1988	WHO, AJCC	PTC	29.85	<0.001 ***	−0.0166	0.981 n.s.	+0.2879 ***	0.001	n.a.	n.a.	n.a.	n.a.
		FTC	1.80	0.1768 n.s.					n.a.	n.a.	n.a.	n.a.
		PTC/FTC gap	22.41	<0.001 ***	−0.0166	0.981 n.s.	+0.2879 ***	0.001	n.a.	n.a.	n.a.	n.a.
1992	AFIP, AJCC	PTC	45.19	<0.0001 ***	+0.6771	0.325 n.s.	+0.2473 ***	<0.001	n.a.	n.a.	n.a.	n.a.
		FTC	2.12	0.1317 n.s.					n.a.	n.a.	n.a.	n.a.
		PTC/FTC gap	35.12	<0.0001 ***	+0.6771	0.325 n.s.	+0.2473 ***	<0.001	n.a.	n.a.	n.a.	n.a.
1997	UICC, AJCC	PTC	79.52	<0.0001 ***	+2.0996 **	0.002	+0.1771 ***	<0.001	+1.0940 ***	<0.001	+0.3010 ***	<0.001
		FTC	2.31	0.1114 n.s.					n.a.	n.a.	n.a.	n.a.
		PTC/FTC gap	68.12	<0.001 ***	+2.0996 **	0.002	+0.1771 ***	<0.001	n.a.	n.a.	n.a.	n.a.
2002	UICC, AJCC	PTC	134.12	<0.0001 ***	+3.7641 ***	<0.001	+0.0175	0.668 n.s.	n.a.	n.a.	n.a.	n.a.
		FTC	3.51	0.0383 *	+0.2337 ***	<0.001	−0.0060	0.167 n.s.	+0.1292 **	0.002	−0.0080 *	0.041
		PTC/FTC gap	102.45	<0.0001 ***	+3.53 ***	<0.001	+0.02	0.540 n.s.	n.a.	n.a.	n.a.	n.a.
2004	WHO	PTC	158.46	<0.0001 ***	+4.1500 ***	<0.001	−0.0736	0.088 n.s.	n.a.	n.a.	n.a.	n.a.
		FTC	4.85	0.0123 *	+0.2106 **	0.001	−0.0104 *	0.036	n.a.	n.a.	n.a.	n.a.
		PTC/FTC gap	142.18	<0.001 ***	+4.1500 ***	<0.001	−0.0736	0.088 n.s.	n.a.	n.a.	n.a.	n.a.
2009	UICC, AJCC	PTC	164.20	<0.0001 ***	+3.7199 ***	<0.001	−0.3218 ***	<0.001	n.a.	n.a.	n.a.	n.a.
		FTC	5.62	0.0066 **	−0.1012	0.171 n.s.	−0.0011	0.885 n.s.	n.a.	n.a.	n.a.	n.a.
		PTC/FTC gap	155.60	<0.0001 ***	+3.7199 ***	<0.001	−0.3218 ***	<0.001	n.a.	n.a.	n.a.	n.a.
2014	AFIP	PTC	72.15	<0.0001 ***	+0.7759	0.451 n.s.	−0.3652 *	0.035	n.a.	n.a.	n.a.	n.a.
		FTC	1.86	0.1672 n.s.					n.a.	n.a.	n.a.	n.a.
		PTC/FTC gap	59.30	<0.001 ***	+0.7759	0.451 n.s.	−0.3652 *	0.035	n.a.	n.a.	n.a.	n.a.
2016	UICC	PTC	41.08	<0.0001 ***	−0.7520	0.520 n.s.	−0.2289	0.354 n.s.	n.a.	n.a.	n.a.	n.a.
		FTC	1.10	0.3418 n.s.					n.a.	n.a.	n.a.	n.a.
		PTC/FTC gap	31.05	<0.0001 ***	−0.7520	0.520 n.s.	−0.2289	0.354 n.s.	n.a.	n.a.	n.a.	n.a.
2017	WHO, AJCC	PTC	29.14	<0.0001 ***	−1.1661	0.361 n.s.	−0.1696	0.587 n.s.	n.a.	n.a.	n.a.	n.a.
		FTC	0.98	0.3831 n.s.					n.a.	n.a.	n.a.	n.a.
		PTC/FTC gap	21.42	<0.001 ***	−1.1661	0.361 n.s.	−0.1696	0.587 n.s.	n.a.	n.a.	n.a.	n.a.

Footnotes: Asterisks denote statistical significance scales (* *p* < 0.05, ** *p* < 0.01, *** *p* < 0.001; n.s. indicates not statistically significant; n.a. (not applicable) labels cells where latent hidden states do not experience active transition differentials.

## Data Availability

The original contributions presented in this study are included in the article and Supplementary Material. Further inquiries can be directed to the corresponding author.

## Supplementary Materials

The following supporting information can be downloaded at: https://www.mdpi.com/article/10.3390/cancers18213468/s1, Figure S1: Chronological Matrix of Histopathology Classifications, Coding Systems, and Registry Data Standards (1974–2023); Table S1: Trends in delay-adjusted SEER incidence rates and USA mortality rates for all cancer sites combined and thyroid cancers by sex.
