# Supplementary material for: Temporal Associations Between Thyroid Cancer Epidemiological Trends and Classification Revisions
Source: Cancers (Basel). 2026 Oct 27;18(21):3468. doi: 10.3390/cancers18213468 (PMC13761937; doi:10.3390/cancers18213468)
Supplement: Supplementary file 1 [file cancers-18-03468-s001.zip › Figure 1 in supplemental material.pdf]

Figure 1 Chronological Matrix of Histopathology Classifications, Coding Systems, and Registry Data Standards (1974–2023)

Data and Breakpoints	Thyroid Classifications	Adesion of SEER	NAACCR Data Standards packages
**Year**	**Thyroid Classifications**	**SEER Program Operations & Guidelines**	NAACCR Data Standards & Packages
**1973**		SEER Program Launch. Commencement of official population-based cancer registration	
1974	◆ WHO | ◆ UICC		
1976		ICD-O-1 Adoption as the Baseline Framework for SEER data collection,	
1977	● AJCC		
1978	◆ UICC		
1983	● AJCC		
1987	◆ UICC		
1988	◆ WHO | ● AJCC	*SEER Program Code Manu* Extent of Disease (EOD)	
1990			
1992	● AFIP | ● AJCC	*SEER Program Code Manual (II)* & ICD-O-2	
1997	◆ UICC | ● AJCC		
1998		*SEER Program Code Manual (III*	
2000			
2001		*SEER Behavior Recode for Analysis*	
2002	◆ UICC | ● AJCC		
2004	◆ WHO	*SEER Program Code Manual (IV)*	
2007		Structural Histology Coding Revision.	Multiple Primary & Histology Rules
2009	◆ UICC | ● AJCC		
2011		ICD-O-3.1 Update (Not uniformly adopted in the USA)	

2014	● AFIP	*SEER Program Coding and Staging Manual 2014*	Guidelines for ICD-O-3 Updates Release
2016	◆ UICC	Solid Tumor Rules / Introduction of NIFTP Context	
2017	◆ WHO | ● AJCC		Finalization of 2018 Standards
2018		Grade & Summary Stage 2018 EOD	ICD-O-3 Update Package
2019			
2021		Annual Thyroid Histology Coding Updates	ICD-O-3.2 Implementation
2022	◆ WHO	Continuous Data Synchronization	ICD-O-3.2 Framework
2023		Standardized Registry Maintenance	ICD-O-3.2 Annual Review

ICD-O (International Classification of Diseases for Oncology)

NAACCR (North American Association of Central Cancer Registries)
