# Supplementary material for: Temporal Associations Between Thyroid Cancer Epidemiological Trends and Classification Revisions
Source: Cancers (Basel). 2026 Oct 27;18(21):3468. doi: 10.3390/cancers18213468 (PMC13761937; doi:10.3390/cancers18213468)
Supplement: Supplementary file 1 [file cancers-18-03468-s001.zip › Legend for Table 1 and Figure 1 of Supplementary material.pdf]

Legend for Table 1 and Figure 1 of Supplementary material

Table 1. Trends in delay-adjusted SEER incidence rates and USA mortality rates for all cancer sites combined and thyroid cancers by sex.

Footnotes.

Data Source: observed SEER Incidence Rate for cancers and U.S. Mortality Rate for cancers are noted. The Annual Percent Change (APC) and direction of APC and Mortality trend are reported. Delay-adjusted SEER Incidence Rate have been noted. This was for ensuring that current cancer trends are not artificially deflated by late-arriving data.

Methodology: rates are per 100,000 and are age-adjusted to the 2000 USA Std Population (20 age groups - Census P25-1130). APC estimates were calculated by using the Joinpoint Trend Analysis Software, Version 6.0, March 2026, National Cancer Institute as reported from from SEER web site, accessed Jul 27, 2026.

The APC's direction is "Rising" when the entire 95% confidence interval (C.I.) is above 0, "Falling" when the entire 95% C.I. is lower than 0, otherwise, the trend is considered "Not Significant".

Abbreviations:

APC: annual percent change estimates %

Figure 1. Chronological Matrix of Histopathology Classifications, Coding Systems, and Registry Data Standards (1974–2023)

Footnotes. Abbreviations: ICD-O (International Classification of Diseases for Oncology) , NAACCR (North American Association of Central Cancer Registries)
