# Supplementary material for: Temporal Associations Between Thyroid Cancer Epidemiological Trends and Classification Revisions
Source: Cancers (Basel). 2026 Oct 27;18(21):3468. doi: 10.3390/cancers18213468 (PMC13761937; doi:10.3390/cancers18213468)
Supplement: Supplementary file 1 [file cancers-18-03468-s001.zip › Table 1 in Supplementary material.pdf]

Table 1. *Trends in delay-adjusted SEER incidence rates and USA mortality rates for all cancer sites combined and thyroid cancers by sex.*

	All cancer sites combined						Papillary and Follicular combined Thyroid Cancers					
	Delay-adjusted SEER Incidence Rate			USA Mortality Rate			Delay-adjusted SEER Incidence Rate			USA Mortality Rate		
**Sex**	**Year Range**	**APC (%)**	**Direction**	**Year Range**	**APC (%)**	**Direction**	**Year Range**	**APC (%)**	**Direction**	**Year Range**	**APC (%)**	**Direction**
**Both Sexes**	2000–2009	-0.2	Not Significant	2000–2009	-1.5	Falling	2000–2009	7.0	Rising	2000–2009	1.2	Rising
	2009–2013	-1.6	Falling	2009–2016	-1.3	Falling	2009–2014	2.1	Rising			
	2013–2023	0.5	Rising	2016–2019	-2.0	Falling	2014–2021	-1.6	Falling			
				2019–2024	-1.4	Falling	2021–2023	5.1	Rising	2009–2024	-0.1	Not Significant
**Female**	2000–2016	0.0	Not Significant	2000–2002	-1.0	Falling	2000–2009	7.1	Rising	2000–2008	1.0	Rising
				2002–2009	-1.5	Falling	2009–2014	2.1	Rising			
				2009–2016	-1.1	Falling	2014–2021	-1.8	Falling	2008–2024	-0.3	Not Significant
	2016–2023	0.9	Rising	2016–2019	-1.8	Falling	2021–2023	5.2	Rising			
				2019–2024	-1.2	Falling						
**Male**	2000–2008	-0.5	Falling	2000–2015	-1.7	Falling	2000–2009	6.6	Rising	2000–2016	1.1	Rising
	2008–2014	-2.2	Falling				2009–2014	2.5	Rising			
	2014–2023	0.5	Rising	2015–2024	-1.8	Falling	2014–2021	-0.8	Not Significant	2016–2024	-0.6	Not Significant
							2021–2023	4.5	Rising			
